# Supplementary material for: Epistasis mediates the role of negative frequency-dependent selection in bacterial strain structure
Source: PLoS Comput Biol. 2026 Mar 19;22(3):e1014083. doi: 10.1371/journal.pcbi.1014083 (PMC13029793; doi:10.1371/journal.pcbi.1014083)
Supplement: S1 File — (PDF) [file pcbi.1014083.s001.pdf]

Supplementary Information for: Epistasis mediates the role of negative frequency-dependent selection in bacterial strain structure

Martin Guillemet[1,2,3*] and Sonja Lehtinen[1,2,3**]

[1]Department of Environmental System Science, Institute for Integrative Biology, ETH Zürich, Zürich, Switzerland
[2]Swiss Institute of Bioinformatics, Lausanne, Switzerland
[3]Department of Computational Biology, University of Lausanne, Lausanne, Switzerland
[*]martin.guillemet@env.ethz.ch
[**]sonja.lehtinen@unil.ch

Supplementary Note A: One metabolic locus with two alleles

In this note we describe explicitly the dynamical equations for all compartments of the model in the case of one metabolic locus with two alleles. In this case efficiency of co-colonisation is only k_0 if the allele of the co-colonising and resident strain is the same or k_1 if the two alleles are different.

$$
\begin{aligned}
\frac{dS}{dt} &= b - Sd - S\beta_0\Big((I_a + I_A) + 2q(I_{a,a} + I_{A,a} + I_{A,A})\Big) + \gamma(I_a + I_A) \\
\frac{dI_a}{dt} &= S\beta_0 I_a + S\beta_0 q(2I_{a,a} + I_{A,a}) \\
&\quad + \gamma(2I_{a,a} + I_{a,A}) - I_a(d+\gamma) \\
&\quad - \beta_0 I_a(2k_1 I_a + k_0 I_A) - \beta_0 I_a q\left(2k_1 I_{a,a} + (k_0 + k_1)I_{a,A} + 2k_0 I_{A,A}\right) \\
\frac{dI_A}{dt} &= S\beta_0 I_A + S\beta_0 q(2I_{A,A} + I_{a,A}) \\
&\quad + \gamma(2I_{A,A} + I_{a,A}) - I_A(d+\gamma) \\
&\quad - \beta_0 I_A(k_1 I_A + k_0 I_a) - \beta_0 I_A q\left(2k_1 I_{A,A} + (k_0 + k_1)I_{a,A} + 2k_0 I_{a,a}\right) \\
\frac{dI_{a,a}}{dt} &= 2\beta_0 I_a^2 k_1 - I_{a,a}(d + 2\gamma) \\
&\quad + \beta_0 q I_a k_1(2I_{a,a} + I_{a,A}) \\
\frac{dI_{A,A}}{dt} &= 2\beta_0 I_A^2 k_1 - I_{A,A}(d + 2\gamma) \\
&\quad + \beta_0 q I_A k_1(2I_{A,A} + I_{a,A}) \\
\frac{dI_{a,A}}{dt} &= 2\beta_0 I_a I_A k_0 - I_{a,A}(d + 2\gamma) \\
&\quad + \beta_0 q\big(I_a k_0(2I_{A,A} + I_{a,A}) + I_A k_0(2I_{a,a} + I_{a,A})\big)
\end{aligned} \tag{1}
$$

Note that recombination terms vanish in this system with only one locus when recombination is symmetric. This would not be the case if recombination was skewed. For instance if allele a and A were respectively the absence and the presence of a gene, one could consider that the gene is preferentially lost rather than acquired, in this case the asymmetric recombination term would remain.

To highlight the negative frequency-dependent selection (NFDS) in this system we introduce

$$
\begin{aligned}
p_A &= \frac{I_A + I_{a,A} + 2I_{A,A}}{I_a + I_A + 2I_{a,a} + 2I_{a,A} + 2I_{A,A}} \\
&= \frac{N_A}{N_{tot}}
\end{aligned} \tag{2}
$$

the frequency of allele A in the bacterial population, with N_A the density of bacteria of strain A and N_{tot} the total density of bacteria. The dynamics of this frequency can be derived as a function of the selection coefficient for allele A which is defined by

$$
w_A(t) = r_A(t) - r_a(t) \tag{3}
$$

where $r_A(t)$ and $r_a(t)$ are respectively the growth rate of bacteria with allele A and a in the population such that

$$
\begin{aligned}
r_A(t) &= \frac{N'_A}{N_A} = \frac{I'_A + 2I'_{A,A} + I'_{a,A}}{I_A + I_{a,A} + 2I_{A,A}} \\
r_a(t) &= \frac{N'_a}{N_a} = \frac{I'_a + 2I'_{a,a} + I'_{a,A}}{I_a + I_{a,A} + 2I_{a,a}}
\end{aligned} \tag{4}
$$

We get:

$$\frac{\mathrm{d}p_A}{\mathrm{d}t} = w_A p_A (1 - p_A) \tag{5}$$

First when $q = 1$, meaning that bacteria in co-colonised hosts are as infectious as bacteria in single-infected hosts (and thus $I_{A,A}$ is twice as infectious as I_A), we get

$$w_A(t) = \beta_0 (k_0 - k_1)(I_a - I_A) \tag{6}$$

We see with this equation when $k_0 > k_1$, which is true in a context of metabolic niche differentiation, that the selection coefficient for allele A is positive when $I_A < I_a$. Hence the less frequent strain will have a higher growth rate than the more frequent strain, and the system is only at stable equilibrium when $I_A = I_a$.

We also derive this selection coefficient for the special value of $q = 1/2$, meaning that a bacteria in a co-colonised host is half as infectious as a bacteria in a single infected host (and so $I_{A,A}$ is equally as infectious as I_A). In this case we get

$$\begin{aligned} w_A(t) &= \frac{1}{2}\beta_0 \Big((k_0 - k_1)(I_a - I_A) + \frac{I_A(S + k_0 I_a + k_1 I_A)}{I_A + I_{a,A} + 2I_{A,A}} - \frac{I_a(S + k_0 I_A + k_1 I_a)}{I_a + I_{a,A} + 2I_{a,a}} \Big) \\ &= \frac{1}{2}\beta_0 \Big((k_0 - k_1)(I_a - I_A) + \frac{I_A(S + k_0 I_a + k_1 I_A)}{N_A} - \frac{I_a(S + k_0 I_A + k_1 I_a)}{N_a} \Big) \end{aligned} \tag{7}$$

Again we find a term like in the previous equation with the difference between the density of both single-infected types which lead to NFDS. The next terms can be seen as the rate of new transmission produced by the single infected of each type, divided by the total density of that strain in the population.

To get a better understanding of these last two terms, one can look at the expression of the selection coefficient when $q = 0$, meaning a strain in co-colonisation is able to transmit:

$$w_A(t) = \beta_0 \Big(\frac{I_A(S + k_0 I_a + k_1 I_A)}{N_A} - \frac{I_a(S + k_0 I_A + k_1 I_a)}{N_a} \Big) \tag{8}$$

In that case we find a very different behaviour (see Fig C). For $q = 0$, only the bacteria in a single infected host can transmit, which means that the rate at which it co-colonises has not direct effect on the selection coefficient i.e. on fitness. However, if only single infected hosts can transmit and only the primary colonisation affects the fitness directly, the driver of the fitness difference between the strains becomes the ability for resident strains to avoid being co-colonised. Thus if the likelihood of being co-colonised increases, fitness decreases. In our metabolic niche framework, a strain is more easily co-colonised by a strain with a different allele. This create a positive frequency-dependent selection on allele frequency: if an allele is in higher frequency, the bacteria carrying this allele will be more likely to evade being co-colonised, and their frequency will increase. Thus going back to the expression of w_A for the more realistic value of $q = 1/2$, we see a combination of the two contrasting effects that are clearly highlighted for the values $q = 0$ or $q = 1$. Fig C highlights how we find a transition between NFDS and PFDS due to these effects for a value close to $q = 0.1$, well below what we consider biologically plausible.

Supplementary Note B: Asymmetric metabolic loci

The metabolic competition model presented in the main text features symmetric alleles at a given locus, which leads to the equilibrium frequencies being equal to 0.5. To explore the effects of a deviation from equal allele frequencies at equilibrium, we extend this model to add an additive benefit to transmission for alleles a and b such that in system (13) the transmission term of genotype x on host i becomes β':

$$\beta'(x, i) = \beta(x, i) + \beta_0 m \tag{9}$$

This additive benefit to transmission ensures that no additional epistasis is generated. The effect on equilibrium allele frequencies and equilibrium LD is shown in Fig H. We find that the effect on allele frequencies of this added benefit to one allele per locus is stronger when k_1 and k_2 get closer to 1, i.e. when the selection imposed by metabolic competition gets weaker. When this allele frequency asymmetry gets very pronounced, we find an impact on equilibrium LD. In the parameter space where epistasis predicts null LD and NFDS on genotype pairs, we find that equilibrium LD can be negative, which is explained by the absence of genotype AB at equilibrium. Indeed, in the top right corner of the heatmaps of Fig H, the added benefit of alleles a and b overwhelms the NFDS imposed by metabolic competition. Going back to Eq (4) of the dynamics of LD, we understand how the effect of epistasis can vanish when a genotype is not able to be maintained: when genotype AB is absent, then $(p_A p_b + D) = 0$, which leads to the term involving epistasis w_{AB} to be zero.

Supplementary Note C: Competition-colonisation with one bi-allelic locus

We now describe explicitly the system of ODEs for the competition-colonisation trade-off model for one bi-allelic locus. In this model, m is the benefit of the coloniser allele a on the primary colonisation. $k_{-1} < k_0 < k_{+1}$ represent the efficiency of co-colonisation depending on the whether the coloniser has respectively one more, the same number, or one less competitive allele A than the resident strain.

$$
\begin{aligned}
\frac{dS}{dt} &= b - dS + \gamma(I_a + I_A) - S\beta_0\Big((1+m)I_a + I_A + q(2(1+m)I_{a,a} + (2+m)I_{a,A} + 2I_{A,A})\Big) \\
\frac{dI_a}{dt} &= \beta_0(1+m)SI_a + \beta_0 Sq\left(2(1+m)I_{a,a} + (1+m)I_{a,A}\right) \\
&\quad + \gamma\left(2I_{a,a} + I_{a,A}\right) - I_a(d+\gamma) \\
&\quad - \beta_0 I_a\left(k_0 I_a + k_{+1} I_A\right) - q\beta_0 I_a\left(2k_0 I_{a,a} + (k_0 + k_{+1})I_{a,A} + 2k_{+1}I_{A,A}\right) \\
\frac{dI_A}{dt} &= \beta_0 SI_A + S\beta_0 q\left(I_{a,A} + 2I_{A,A}\right) \\
&\quad + \gamma\left(I_{a,A} + 2I_{A,A}\right) - I_A(d+\gamma) \\
&\quad - \beta_0 I_A\left(k_{-1} I_a + k_0 I_A\right) - q\beta_0 I_A\left(2k_{-1} I_{a,a} + (k_{-1} + k_0)I_{a,A} + 2k_0 I_{A,A}\right) \\
\frac{dI_{a,a}}{dt} &= \beta_0 k_0 I_a^2 + q\beta_0 k_0 I_a\left(2I_{a,a} + I_{a,A}\right) - (d+2\gamma)I_{a,a} \\
\frac{dI_{A,A}}{dt} &= \beta_0 k_0 I_A^2 + q\beta_0 k_0 I_A\left(I_{a,A} + 2I_{A,A}\right) - (d+2\gamma)I_{A,A} \\
\frac{dI_{a,A}}{dt} &= \beta_0(k_{-1} + k_{+1})I_a I_A - (d+2\gamma)I_{a,A} \\
&\quad + q\beta_0\left[I_A\left(2k_{-1}I_{a,a} + k_{-1}I_{a,A}\right) + I_a\left(k_{+1}I_{a,A} + 2k_{+1}I_{A,A}\right)\right]
\end{aligned}
\tag{10}
$$

With the same reasoning as the previous section, we get for the case $q = 1$ the expression for the selection coefficient of allele A as:

$$
w_A(t) = \beta_0\left((k_{+1} - k_0)\, I_0(t) + (k_0 - k_{-1})\, I_1(t) - mS(t)\right) \tag{11}
$$

Therefore, selection favours the coloniser allele a when the number of uncolonised hosts S is high compared to the number of single colonised hosts I_a and I_A. However, when the coloniser allele increases in frequency, there is a stronger pressure and thus a depletion of S hosts compared to single colonised hosts. In turn, this favours the selection for the competitive allele, thus leading to balancing selection. However, the equilibrium frequency of each allele is not necessarily 0.5, and diversity is not necessarily maintained for all sets of parameters. We highlight this in Fig Ja and Jb, showing how the equilibrium frequency of the competitive allele A increases with k_{+1} and decreases with m or k_{-1}.

Similarly to the previous model, we also explore the case where $q = 0.5$, meaning that the efficiency of colonising from a co-colonised host is reduced by half compared to a single colonised host. The effect on the selection coefficient is

$$
\begin{aligned}
w_A(t) = \frac{\beta_0}{2}\Big((k_{+1} - k_0)\, I_0(t) + (k_0 - k_{-1})\, I_1(t) - mS(t) \\
+ \frac{I_A(S + k_{+1} I_a + k_0 I_A)}{N_A} - \frac{I_a(S + k_{-1} I_A + k_0 I_a)}{N_a} \Big)
\end{aligned}
\tag{12}
$$

Balancing selection therefore emerges as the density of uncolonised hosts reduces the selection coefficient for the competitive genotype A, which depletes the S population less than the coloniser a. The impact on the frequency of the competitive allele is presented in Fig Jc and Jd.

Supplementary Note D: Exploring the outcomes of the competition-colonisation model

To explore the outcomes of the competition-colonisation model (Fig K and L), we use two meta-parameters to explore different geometries for the competition efficiencies k_Δ. We use a concavity parameter p_c and a step parameter p_s, which we implement as:

$$
\begin{aligned}
k_{-1} &= logistic(logit(0.25) + p_c + 0.5p_s) \\
k_0 &= logistic(logit(0.5) + p_c - p_s) \\
k_{+1} &= logistic(logit(0.75) + p_c + 0.5p_s)
\end{aligned}
\tag{13}
$$

using the logistic and logit functions defined as :

$$
\begin{aligned}
logistic(x) &= \frac{1}{1 + e^{-x}} \\
logit(x) &= Log(\frac{x}{1 - x})
\end{aligned}
\tag{14}
$$

The effects of these parameters on the geometries of the k parameters and on equilibrium LD are shown both in Fig K and L respectively in the absence and presence of recombination. For a step parameter of 0, the concavity parameter dictates whether the resulting geometry of competition parameters k_Δ is either concave or convex, therefore dictating whether competitive genes act antagonistically or synergistically. The step parameter is less straight-forward: it adds a deviation from pure convexity or pure concavity and makes the geometry more rugged, creating a step-like geometry.

It is worth noting that we introduce these meta-parameters in order to explore and visualise the model's behaviour in parameter space. While the meta-parameters are tricky to interpret and measure, the k parameters themselves have clear interpretations and are measurable.

Supplementary Note E: On the asymmetry of comparing LD changes depending on the focal population

We observed in Table 2 that the odds-ratio for LD conservation in the observed versus simulated datasets are asymmetric depending on which population is focal (eg. from MaeLa to Massachusetts or from Massachusetts to MaeLa). In this note, we give a likely explanation for this for pairs that involve the MaeLa population.

As observed in Fig P, there are more pairs of genes in high D' in the Massachusetts and Southampton population than in the MaeLa population. This is likely due to the higher diversity observed in the MaeLa dataset (see how it encompasses more of the phylogenetic tree than the two other populations

in Fig M): if there are very few "strains" in a population, then a lot of strong LD can be generated stochastically. The most extreme illustration of this being a population with two strains, with no accessory genome diversity within strain, where a pair of gene would have 1/4 chances to have $|D'| = 1$ and 1/4 chances to have $|D'| = -1$. This would be much less likely with more strains. Note that this is specifically not an effect the sample size, see Fig U which reproduces the results of Fig 4 in Main Text subsampling all populations to the size of the Southampton dataset.

Now, when we measure the proportion of conservation between two simulated populations, we only obtain conservation due to chance and the phylogenetic structure of the populations. In the observed dataset, we also detect this stochastic conservation. However we also detect potentially "real" conservation actually caused by selection and epistasis.

Therefore, the odds-ratio we measure can actually be seen as the ratio between the selective and stochastic conservation events divided by the stochastic conservation events. The detection of real, selective conservation events would be independent of which population are chosen as focal or target. However this is not the case for the stochastic conservation events: if there are more high LD pairs in pop2 than pop1, then by chance, a high LD pair in pop1 is more likely to be conserved in pop2 than the other way around. In our dataset, this means that there are more stochastic conservation events when Massachusetts or Southampton (high general LD) are the target population than when MaeLa is (low general LD). Finally, this leads to the ratio of (selective+stochastic)/(stochastic) being expectedly higher when MaeLa is the target population (and Southampton or Massachusetts are the focal populations) than *vice versa*.

Finally, this explanation would predict that the conservation odds-ratio should necessarily be positive. However, we remove a lot of LD conservation by applying the distance threshold of 100kb. For instance, genes locked together in an operon and consistently in high positive linkage are removed from our analyses.

Table A. Significance of LD Conservation across populations and time (All Pairs, Two-Sided Empirical Test)

Comparison	Focal Population	N Pairs Observed	Observed Prop. Cons.	Simulated Prop. Cons.	Odds Ratio	p-value
MaeLa ↔ South	MaeLa	123 640	0.3973	0.3198	**1.24**	< 0.001
	South	206 190	0.2383	0.0601	**3.96**	< 0.001
Mass ↔ MaeLa	Mass	188 646	0.2627	0.0746	**3.52**	< 0.001
	MaeLa	123 640	0.4007	0.2837	**1.41**	< 0.001
Mass ↔ South	Mass	188 646	0.5343	0.4260	**1.25**	< 0.001
	South	206 190	0.4888	0.3047	**1.60**	< 0.001
Pre- ↔ Post-vaccine	2007	275 204	0.4606	0.3704	**1.24**	< 0.001
	2011	281 200	0.4507	0.3583	**1.26**	< 0.001

The table shows linkage analysis results between population pairs using a two-sided empirical test on **all gene pairs** (no distance filtering). Columns represent: compared populations, which focal population was analysed (Focal Population), number of strong linkage pairs analysed in the observed datasets (N Pairs Observed), observed and simulated proportions of conservation events (Prop. Cons.) and p-values. The simulated conservation events proportion is the average over 1000 simulations. Odds-ratios over 1 are shown in bold.

Table B. Significance of LD Reversal across populations and time (All Pairs, Two-Sided Empirical Test)

Comparison	Focal Population	N Pairs Observed	Observed Prop. Rev.	Simulated Prop. Rev.	Odds Ratio	p-value
MaeLa ↔ South	MaeLa	123 640	0.0888	0.0232	**3.83**	< 0.001
	South	206 190	0.0533	0.0044	**12.23**	< 0.001
Mass ↔ MaeLa	Mass	188 646	0.0213	0.0032	**6.61**	< 0.001
	MaeLa	123 640	0.0324	0.0122	**2.65**	< 0.001
Mass ↔ South	Mass	188 646	0.0098	0.0078	**1.26**	0.540
	South	206 190	0.0090	0.0056	**1.61**	0.118
Pre- ↔ Post-vaccine	2007	275 204	0.0306	0.0416	0.74	0.078
	2011	281 200	0.0300	0.0402	0.75	0.092

The table shows linkage analysis results between population pairs using a two-sided empirical test on **all gene pairs** (no distance filtering). Columns represent: compared populations, which focal population was analysed (Focal Population), number of strong linkage pairs analysed in the observed datasets (N Pairs Observed), observed and simulated proportions of reversal events (Prop. Rev.) and p-values. The simulated reversal events proportion is the average over 1000 simulations. Odds-ratios over 1 are shown in bold.

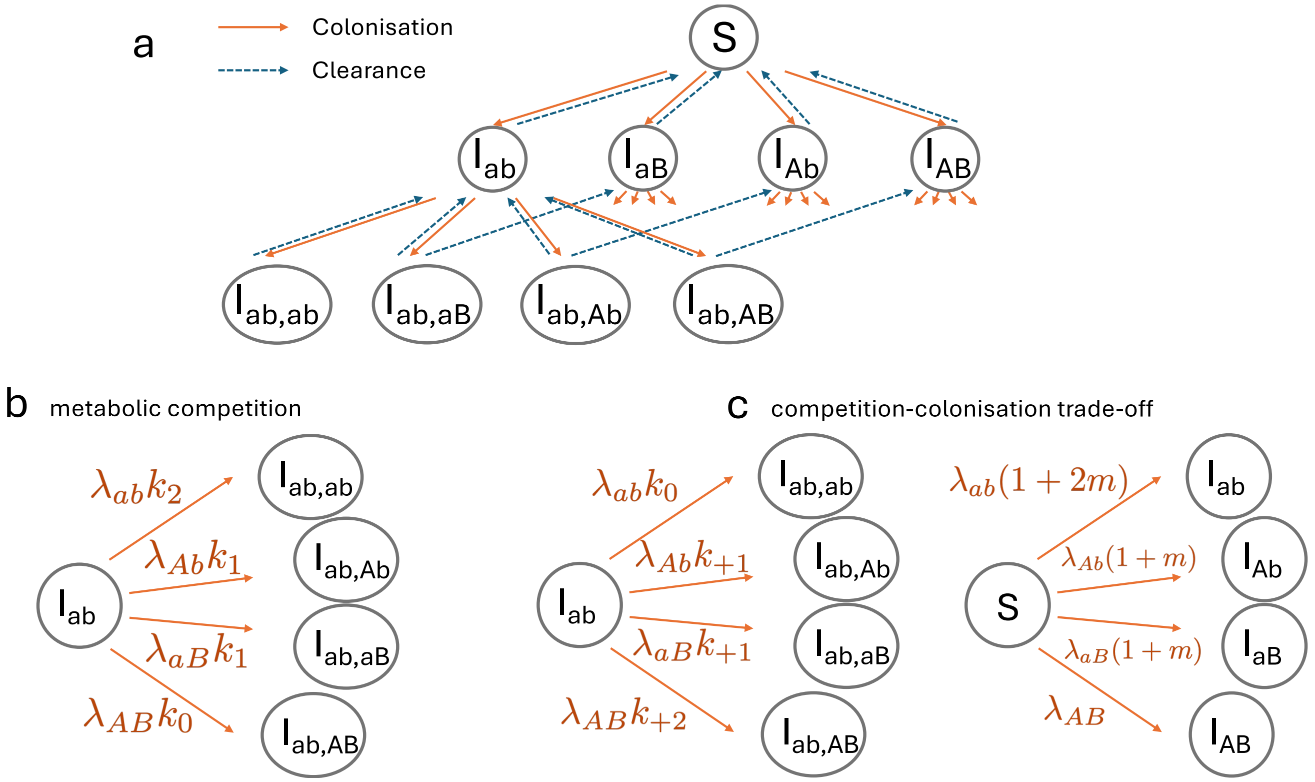

Fig A. Co-colonisation framework with two bi-allelic loci. (a) We represent a schematic of the co-colonisation framework we use throughout this work. Hosts can be uncolonised (S), single colonised (I_x) or co-colonised ($I_{x,y}$). Hosts can be co-colonised by the same strain twice. In the metabolic niche model (b), co-colonisation rates are reduced when the resident and incoming strains share the same allele, modeled by parameters $k_2 < k_1 < k_0$. We show the rates of colonisation for a focal host already colonised by strain ab. In the competition-colonisation model (c), lower case alleles are "colonising", leading to an increased rate of primary colonisation m, while the competitive upper case alleles leads to higher rates of co-colonisation on average. Co-colonisation rates are dependent on the difference in number of competitive alleles between the incoming and the resident strain Δ through the parameters k_Δ. Again, we show the rates of colonisation for a focal host already colonised by strain ab. $\lambda_{ab} = \beta_0(I_{ab} + q(I_{ab,ab} + \sum_{j\epsilon G} I_{ab,j})$ represents the partial force of infection of genotype ab, taking into account that strains in co-colonisation have reduced transmission efficiency through parameter q. Solid orange lines represent colonisation and dashed blue lines represent clearance.

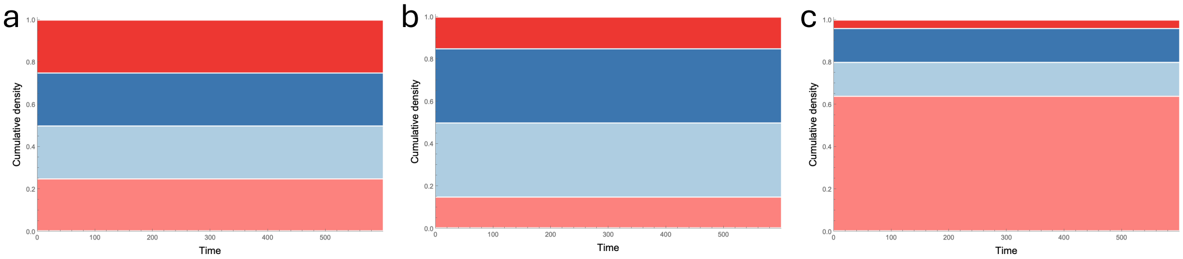

Fig B. The metabolic competition model is structurally neutral. We explore the model in a neutral case: $k_0 = k_1 = k_2 = 1$ for three sets of initial conditions: (a) $D = 0, f_A = f_B = 0.5$, (b) $D = -0.1, f_A = f_B = 0.5$, (c) $D = 0, f_A = f_B = 0.8$, and find that the frequencies and LD stay constant. Parameter values used: $\beta_0 = 2$, $b = 4$, $\gamma = 2$, $d = 1$, $k_0 = 1$, $q = 1$.

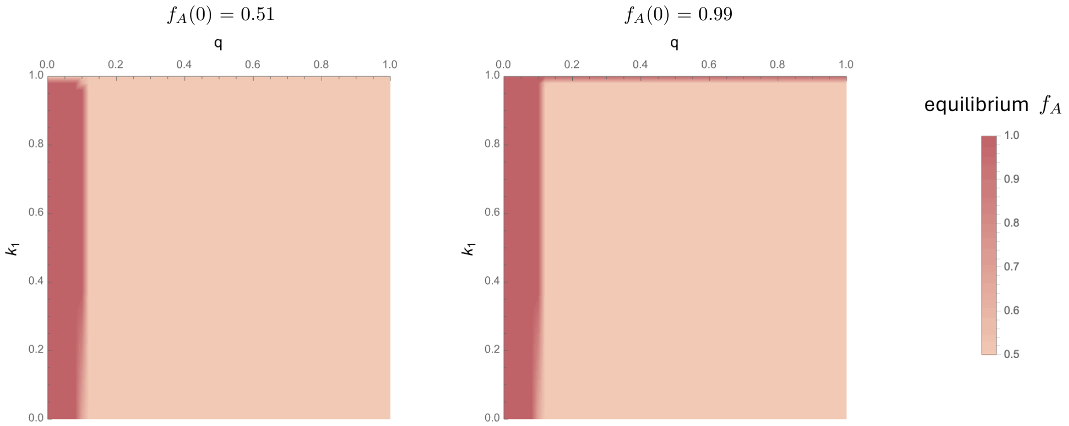

Fig C. Equilibrium frequency in the one locus metabolic niche model as a function of competition parameter k_1 and efficiency of transmitting from a co-colonised host q, for different starting frequencies p_A. Note that the behaviour for a starting frequency $p_A < 0.5$ is symmetric, due to the symmetry of alleles a and A. Parameter values used: $\beta_0 = 2$, $b = 4$, $\gamma = 2$, $d = 1$, $k_0 = 1$, $q = 0.5$.

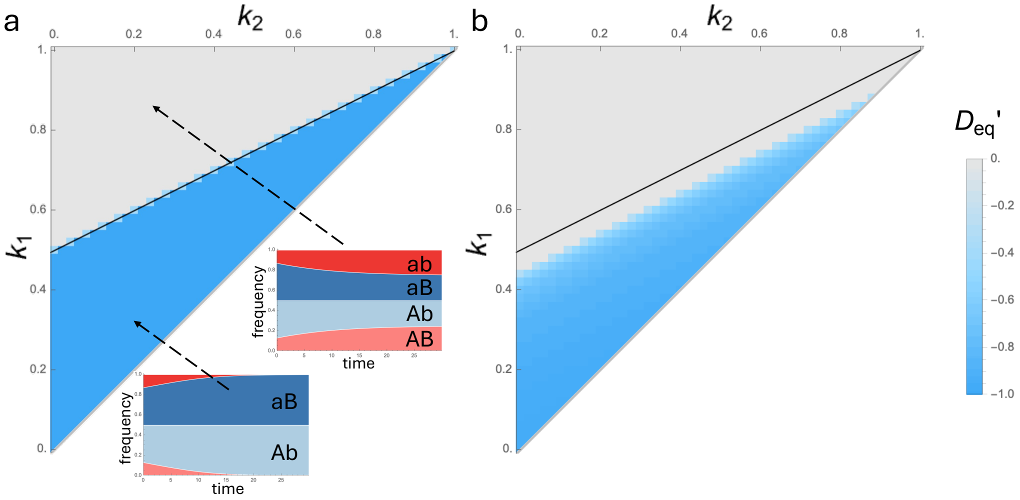

Fig D. Equilibrium LD is null or negative when starting with an initially negative LD. This figure reproduces the results of Fig 2 but with a negative starting value of linkage disequilibrium $D'(0)$. Müller plots in panel (a) show the dynamics of the cumulative frequencies of all genotypes through time, depending on the competition parameters, starting for an initial LD of $D'(0) = -0.4$. Parameter values used: $\beta_0 = 2$, $b = 4$, $\gamma = 2$, $d = 1$, $k_0 = 1$, $q = 0.5$, $\sigma \in \{0, 0.02\}$.

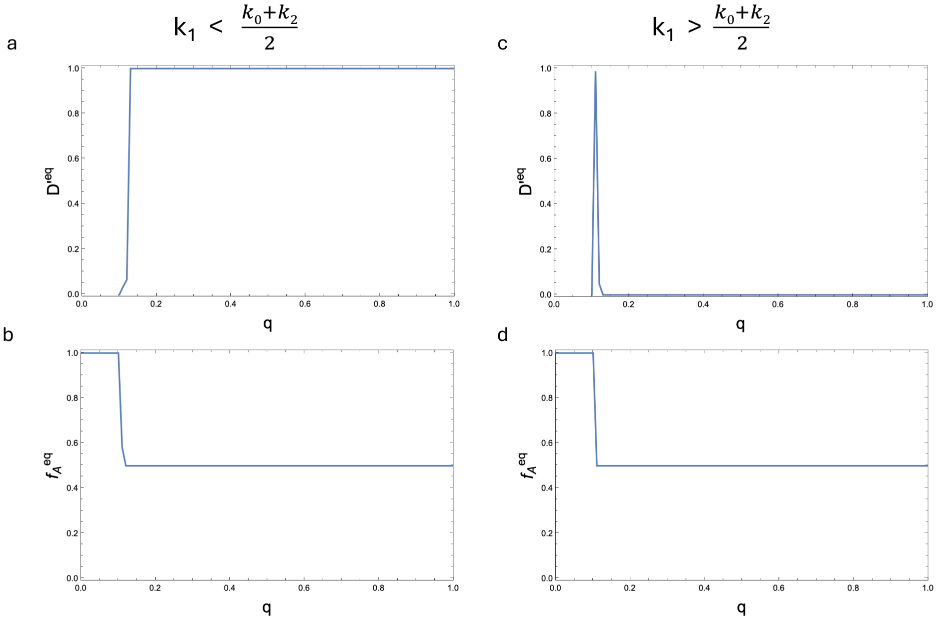

Fig E. Equilibrium LD (a,c) and allele frequency (b,d) in the metabolic niche model as a function of q. Values of equilibrium D' are not shown when $f_A = 1$ because it is not defined. Initial LD is $D' = 0.2$. We chose initial values of frequencies $f_A(0) = f_B(0) = 0.5$. For panels (a,b): $k_2 = 0.2, k_1 = 0.5, k_0 = 1$, or panels (c,d): $k_2 = 0.2, k_1 = 0.7, k_0 = 1$. Other parameter values used: $\beta_0 = 2$, $b = 4$, $\gamma = 2$, $d = 1$.

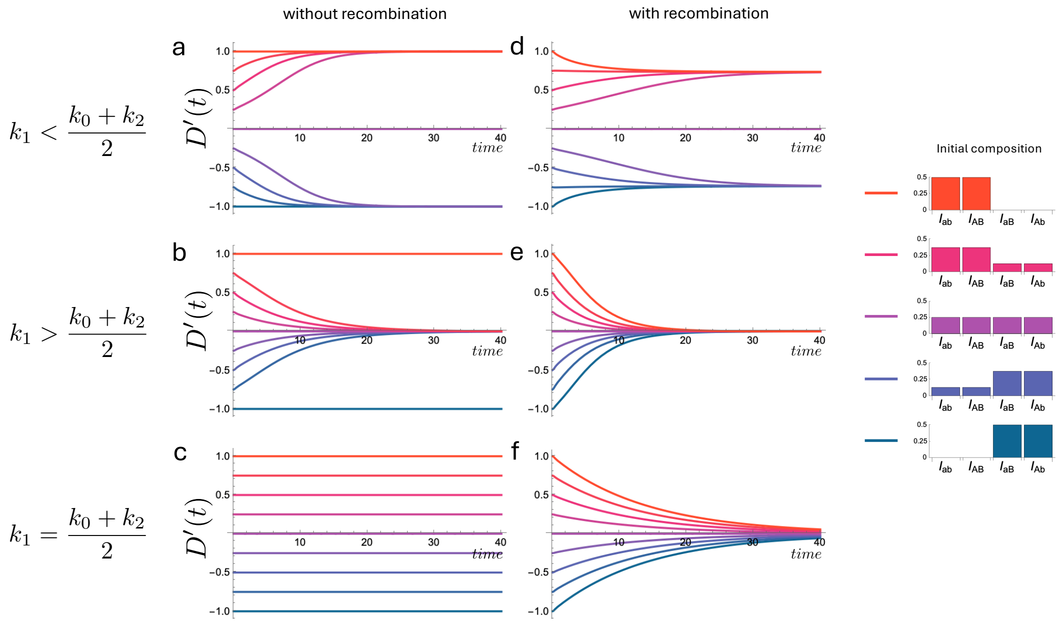

Fig F. Linkage disequilibrium trajectories in the two different competitive regimes of the metabolic niche model: NFDS or PFDS. The values of LD are shown through time for 5 different initial population compositions, shown on the right, characterised by values of initial LD of $D' \in \{-1, -0.5, 0, 0.5, 1\}$. For panels (a,d): $k_2 = 0.2, k_1 = 0.5, k_0 = 1$, for panels (b,e): $k_2 = 0.2, k_1 = 0.7, k_0 = 1$, for panels (c,f): $k_2 = 0.2, k_1 = 0.6, k_0 = 1$, and for panels (b,d) $\sigma = 0.05$. Other parameter values used: $\beta_0 = 2$, $b = 4$, $\gamma = 2$, $d = 1$, $q = 0.5$, $\sigma \in \{0, 0.02\}$.

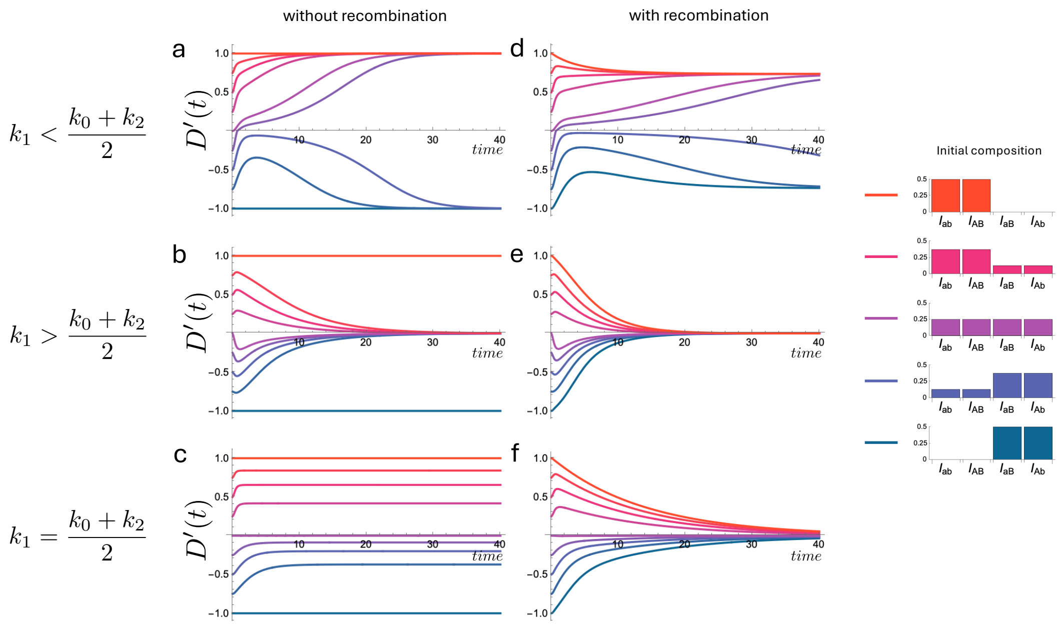

Fig G. Linkage disequilibrium trajectories with asymmetric starting frequencies. The values of LD are shown through time for 5 different initial population compositions, shown on the right, characterised by values of initial LD of $D' \in \{-1, -0.5, 0, 0.5, 1\}$, while for both loci the allele frequencies are $f_A = f_B = 0.1$. For panels (a,d): $k_2 = 0.2, k_1 = 0.5, k_0 = 1$, for panels (b,e): $k_2 = 0.2, k_1 = 0.7, k_0 = 1$, for panels (c,f): $k_2 = 0.2, k_1 = 0.6, k_0 = 1$, and for panels (b,d) $\sigma = 0.05$. Other parameter values used: $\beta_0 = 2$, $b = 4$, $\gamma = 2$, $d = 1$, $q = 0.5$, $\sigma \in \{0, 0.02\}$.

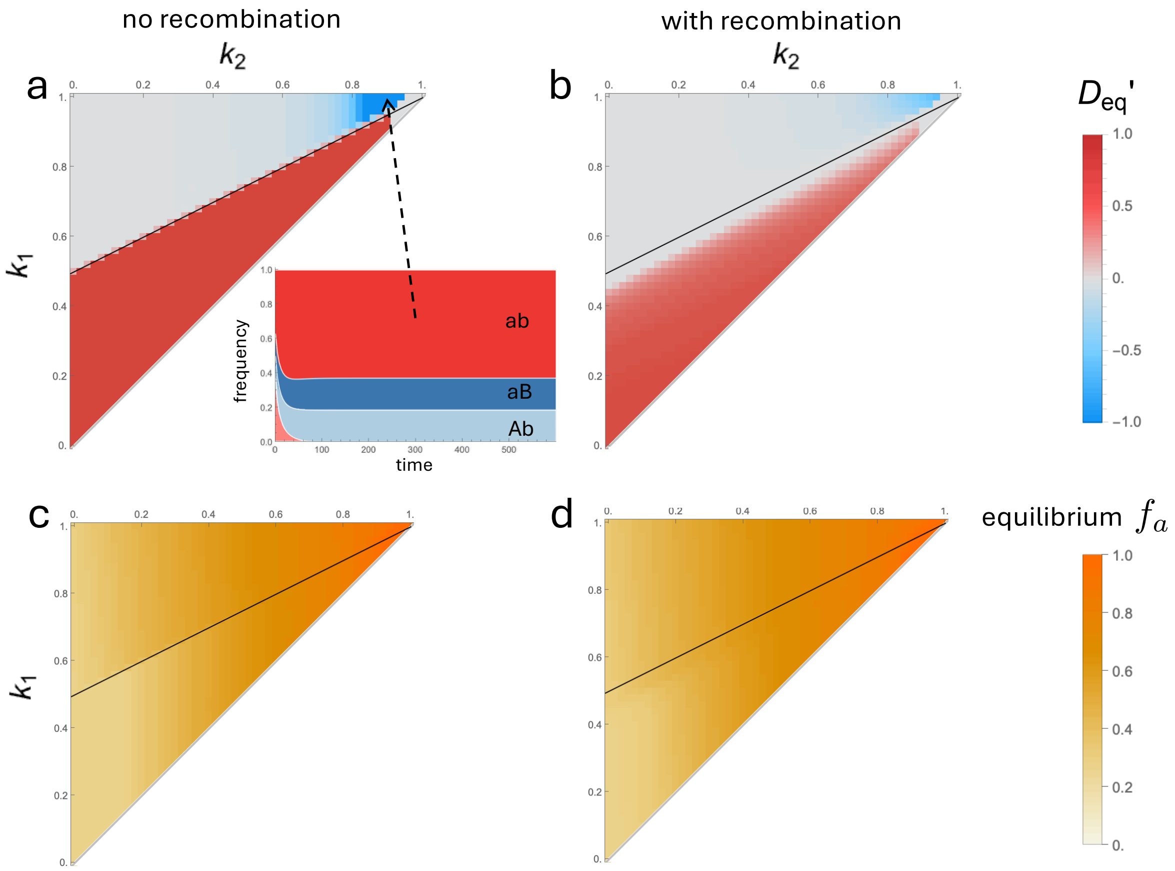

Fig H. Equilibrium LD and allele frequency with allele fitness asymmetry. This figure reproduces the results of Fig 2 in the case where alleles a and b confer an additive transmission benefit $\beta_0\, m_{ab}$. Panels (a,b) show the equilibrium LD respectively without and with recombination for an initial LD of $D'(0) = 0.4$.Panels (c,d) show the corresponding equilibrium frequency of alleles a (and therefore allele b as they are identical). The Müller plot in panel (a) show the dynamics of the cumulative frequencies of all genotypes through time, for $k_1 = 0.99$ and $k_2 = 0.9$. Parameter values used: $\beta_0 = 2$, $b = 4$, $\gamma = 2$, $d = 1$, $k_0 = 1$, $q = 1$, $m_{ab} = 0.03$, $\sigma \in \{0, 0.02\}$.

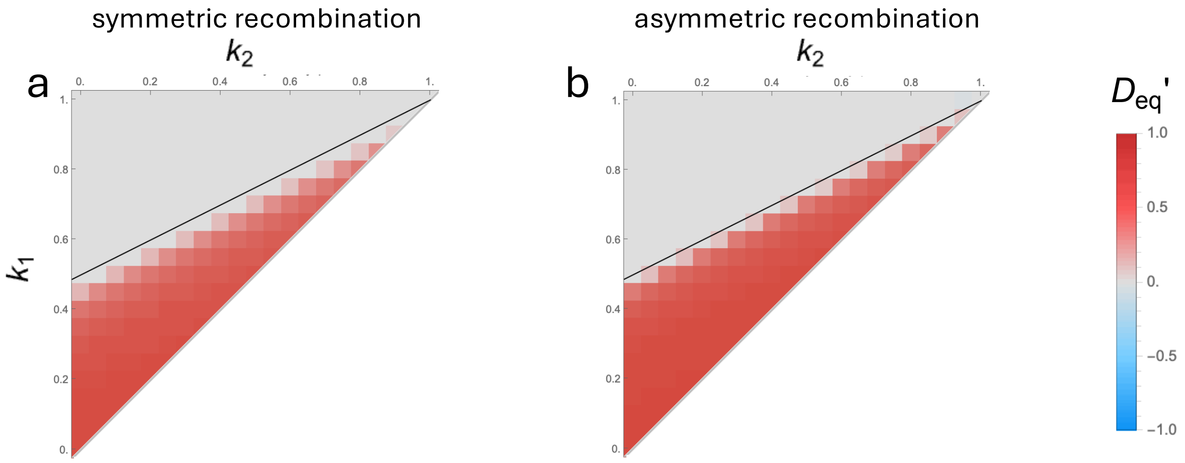

Fig I. Equilibrium LD and allele frequency with symmetric or asymmetric recombination. This figure reproduces the results of Fig 2 comparing a (a) symmetric recombination and (b) asymmetric recombination with parameter $\phi = 0.05$. We use this parameter consistent with Harrow et al. [1], where ϕ denotes the probability that if the donor strain has allele A (resp. B) and the receiving strain has allele a (resp. b), then there is a probability ϕ that the receiving strain actually gets the new allele. Other cases of recombination are unchanged (i.e. donor has a (resp. b) and receiver A (resp. B), or same allele for donor and receiver). This models the known fact that gene deletion is much more common than acquisition for *Streptococcus pneumoniae* [1] . Panels (a,b) show the equilibrium LD respectively without and with recombination for an initial LD of $D'(0) = 0.4$. Parameter values used: $\beta_0 = 2$, $b = 4$, $\gamma = 2$, $d = 1$, $k_0 = 1$, $q = 1$, $\sigma = 0.02$, $\phi \in \{0.05, 1\}$.

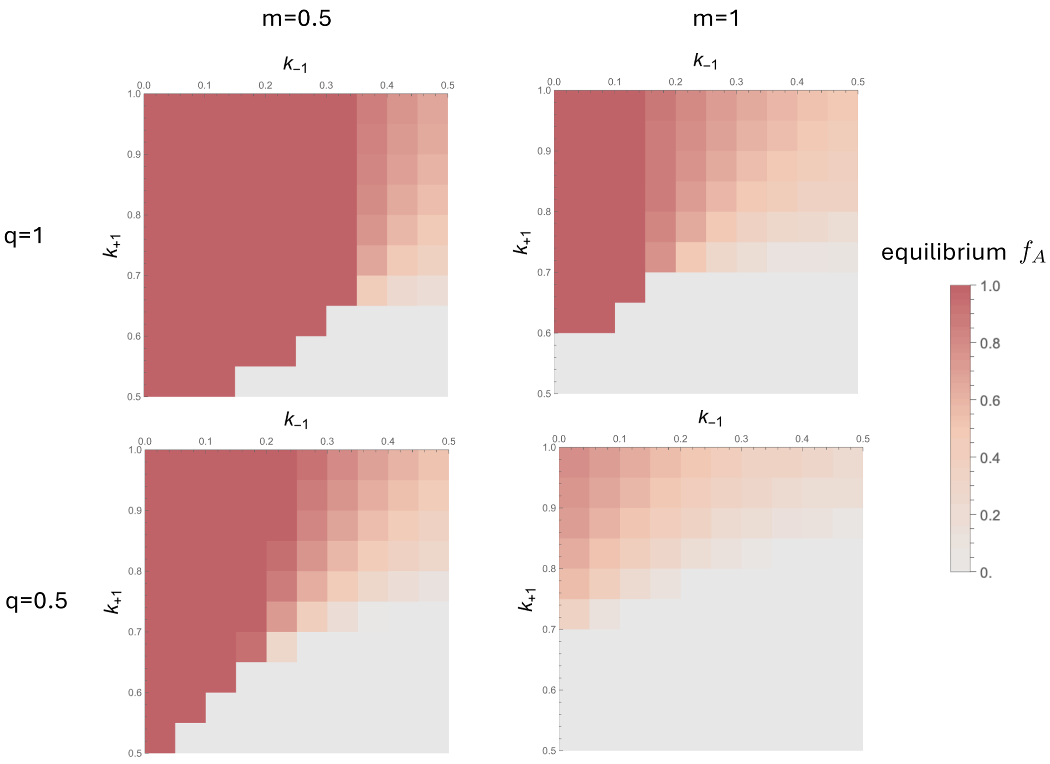

Fig J. Equilibrium frequency of the competitive allele A in the competition-colonisation model according to the competitive efficiencies k_{-1} and k_{+1}, with different values of q the efficiency of colonisation from a co-colonised host and the benefit of the colonising allele m. Parameter values used: $\beta_0 = 2$, $b = 4$, $\gamma = 2$, $d = 1\,\sigma = 0$, $k_0 = 0.5$.

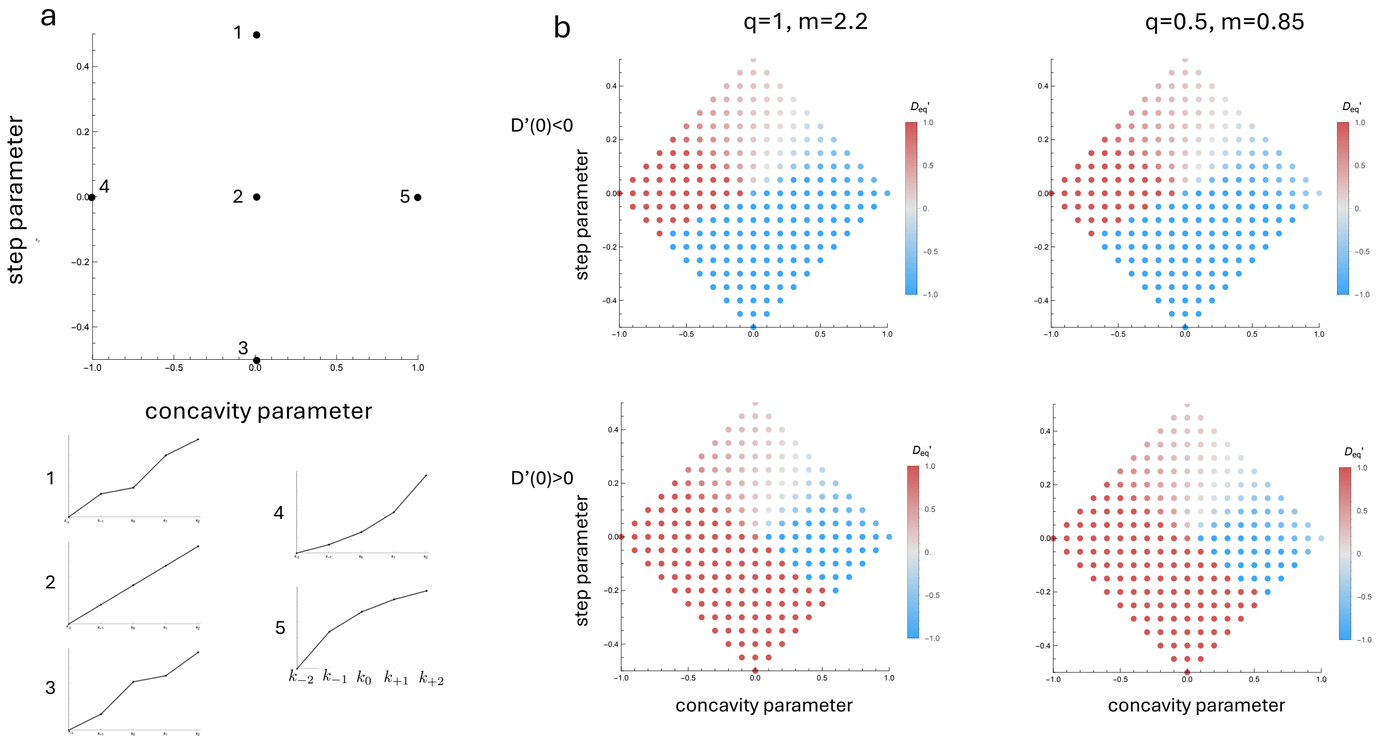

Fig K. Strain structuring effects of a competition-colonisation trade-off model. The outcomes of the models are explored according to two meta-parameters that control the geometry of the competition efficiencies. In (a), the geometry on the competition efficiencies of the concavity and a step parameters are given for five specific combination of values (see Methods, Eq (13)). (b) Equilibrium D' is shown as a function of the step and concavity parameters starting from a negative (first row) or a positive (second row) value of linkage disequilibrium $D'(0)$. The two columns show the results for two values of efficiency from a co-colonised host q and colonising benefit m. The value of m was chosen so that in the additive case, the frequency of each allele was close to 0.5. Recombination is absent. For the case with recombination, see Fig L. Parameter values used: $\beta_0 = 2$, $b = 4$, $\gamma = 2$, $d = 1$ $\sigma = 0$.

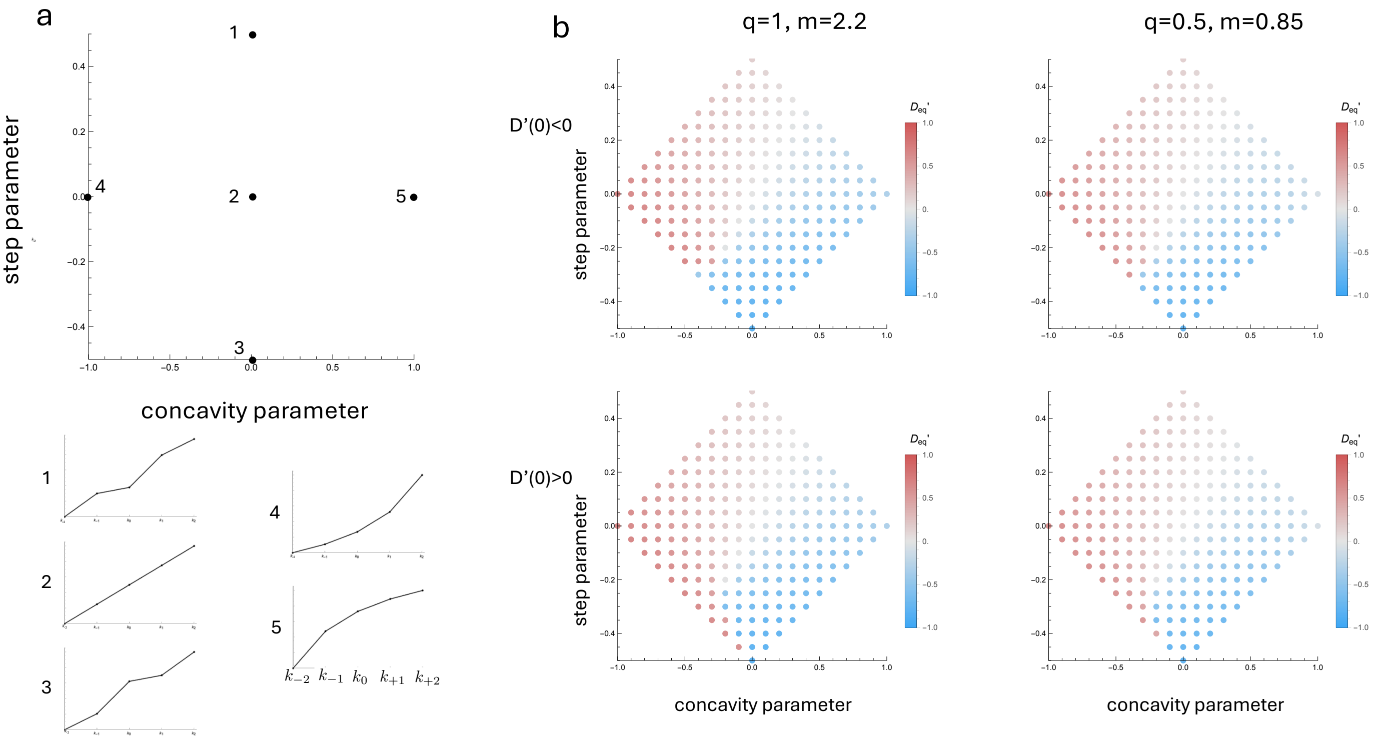

Fig L. Strain structuring effects of a competition-colonisation trade-off model in the presence of recombination. The outcomes of the models are explored according to two meta-parameters that control the geometry of the competition efficiencies. In (a), the geometry on the competition efficiencies of the concavity and a step parameters are given for five specific combination of values(see Methods, Eq (13)). (b) Equilibrium D' is shown as a function of the step and concavity parameters starting from a negative (first row) or a positive (second row) value of linkage disequilibrium $D'(0)$. The two columns show the results for two values of efficiency from a co-colonised host q and colonising benefit m. The value of m was chosen so that in the additive case, the frequency of each allele was close to 0.5. Parameter values used: β_0 = 2, b = 4, γ = 2, d = 1, σ = 0.05.

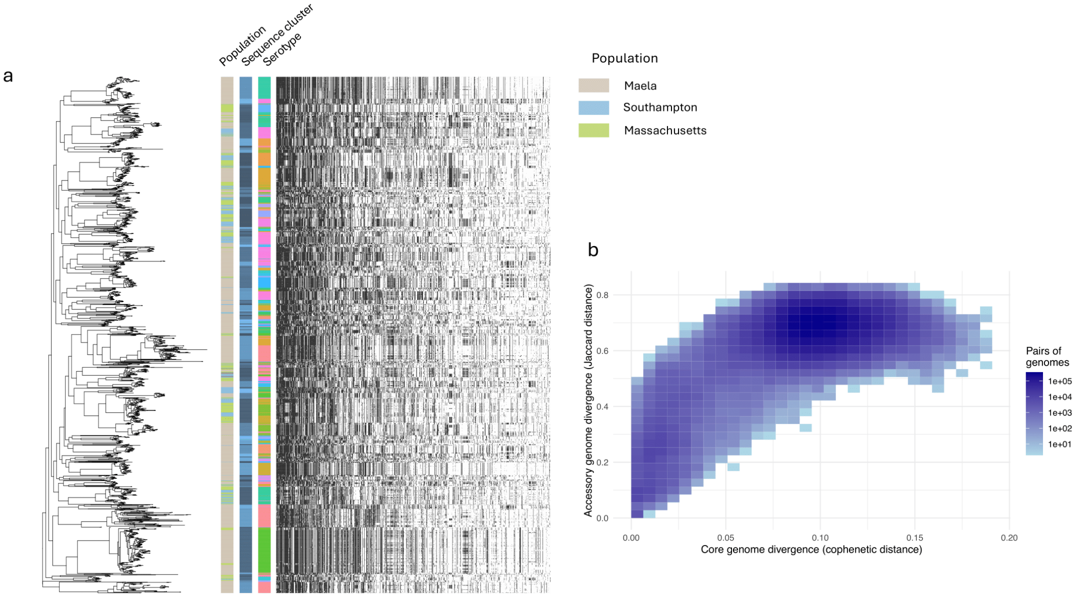

Fig M. The strain structure of *Streptococcus pneumoniae*. (a) Distribution of sequence clusters, serotypes and accessory genes on the phylogenetic tree of *Streptococcus pneumoniae*. Different colours indicate different sequence clusters and serotypes. Accessory genes are sorted left to right from most frequent to less frequent. Only genes with frequency between 0.1 and 0.9, and with more than 30 gains and losses throughout the tree are shown. (b) Gene content relatedness as a function of phylogenetic distance for all pairs of genomes.

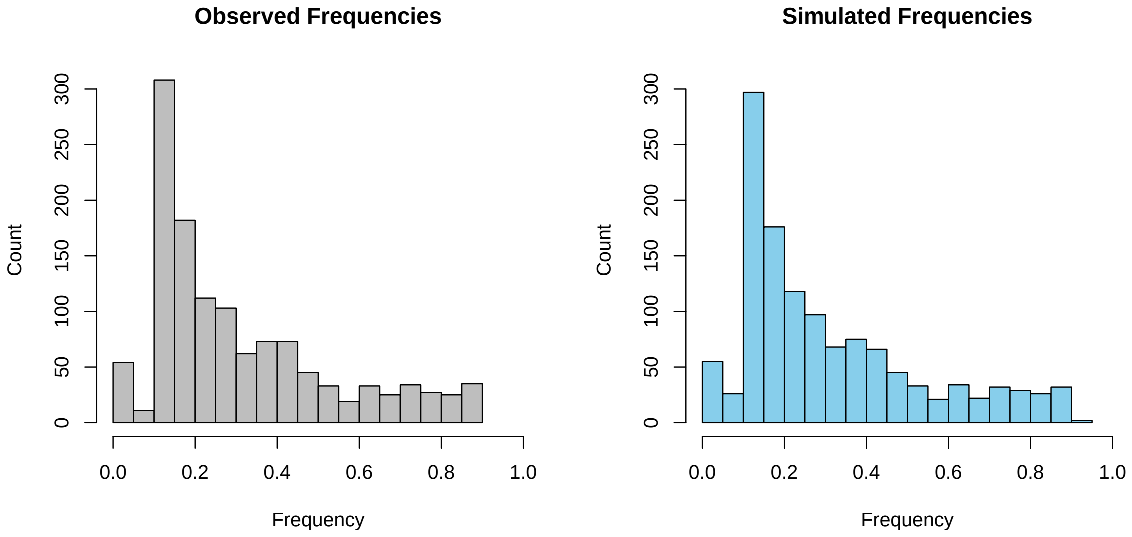

Fig N. Accessory gene frequency distributions in the observed dataset and in a simulated population.

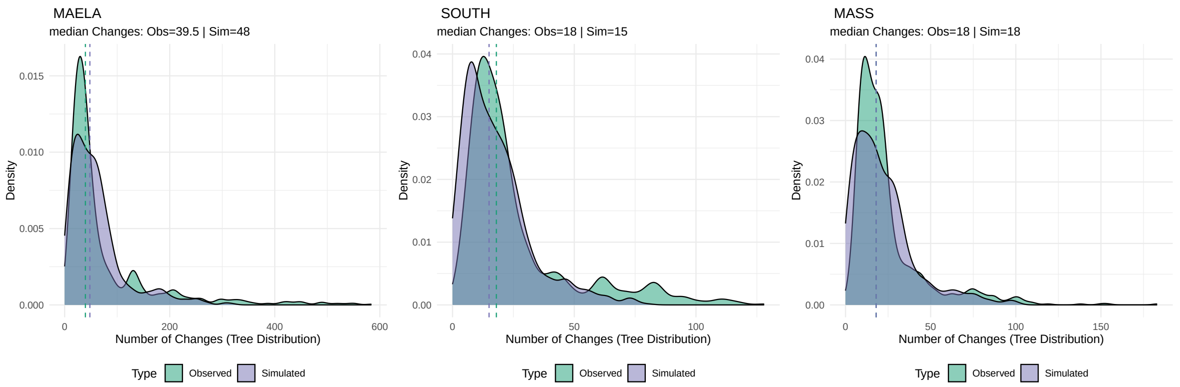

Fig O. Comparison of the distributions of number of gains and losses on the tree (maximum parsimony scores) in the observed (green) and simulated (blue) populations. The median of each distribution are shown, as they were used to calibrate the scaling factor F for the simulations

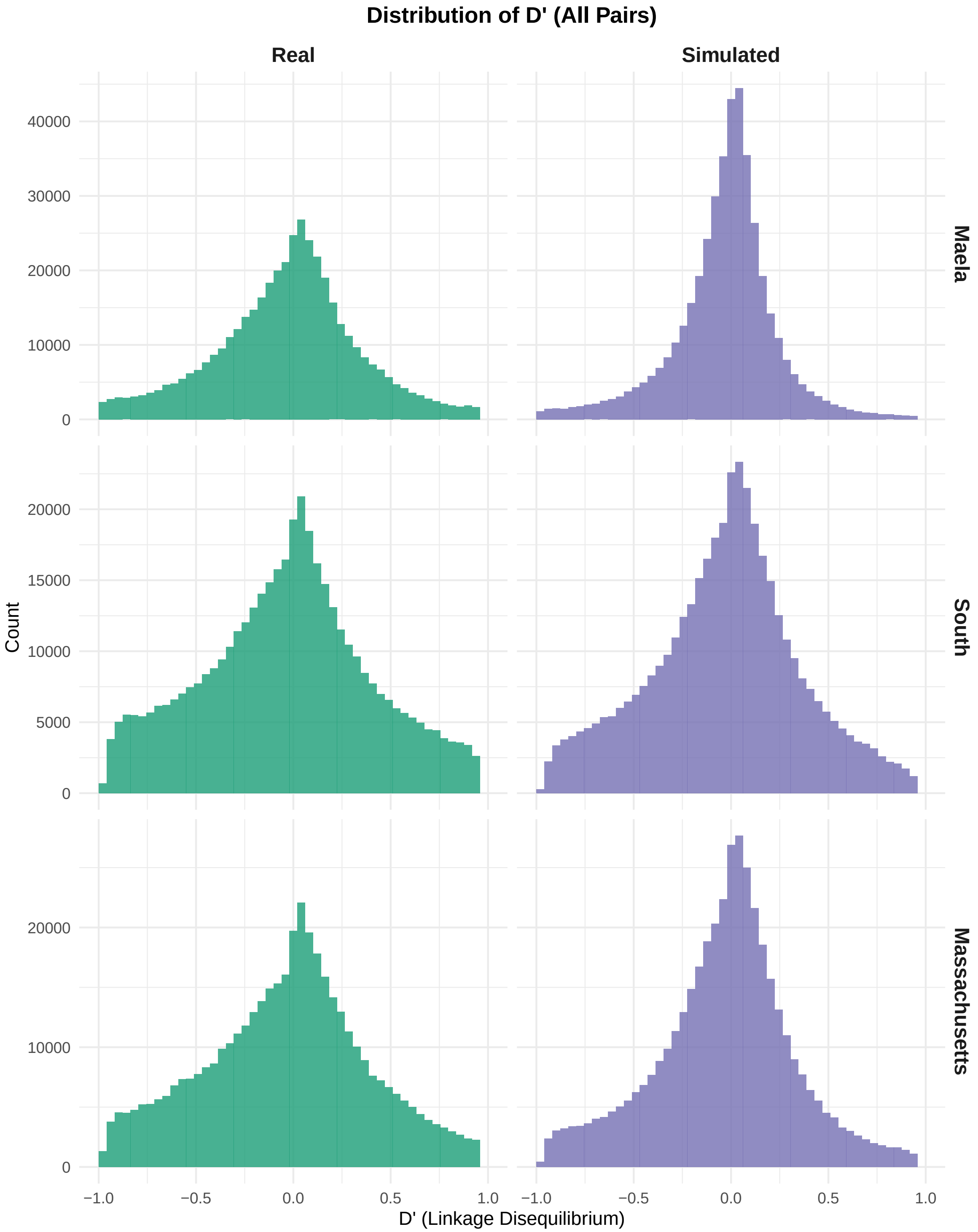

Fig P. Linkage disequilibrium distribution in all populations for the observed and simulated datasets.

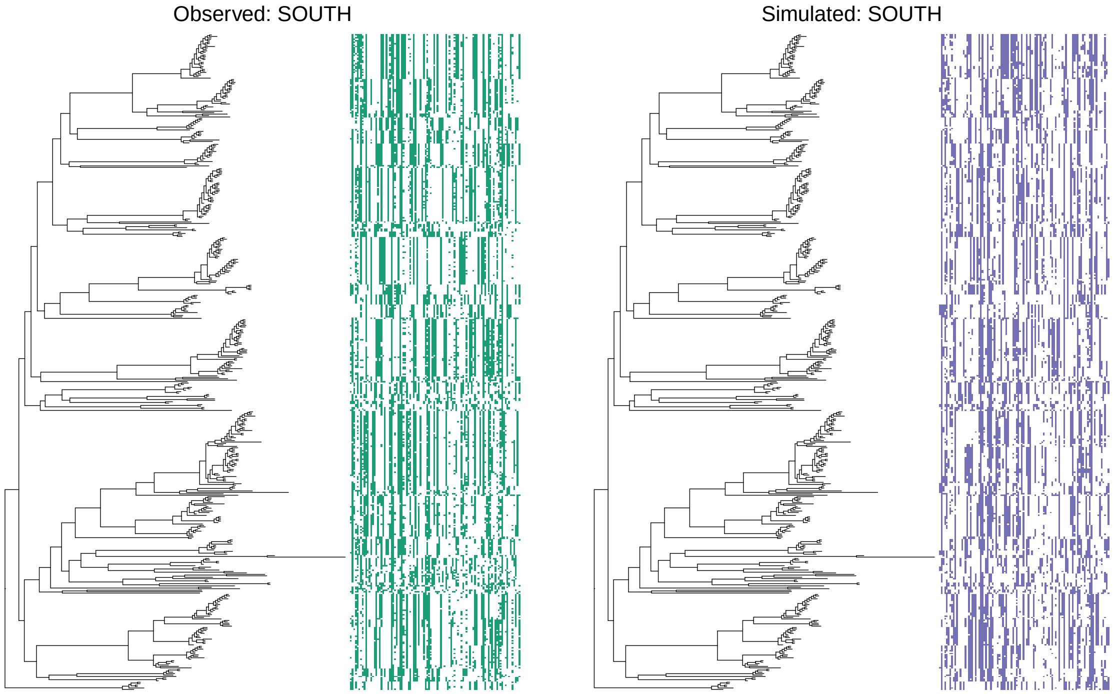

Fig Q. Gene distribution in the Southampton population for both the observed and simulated datasets. A colored cell represents presence and white represents absence of the gene.

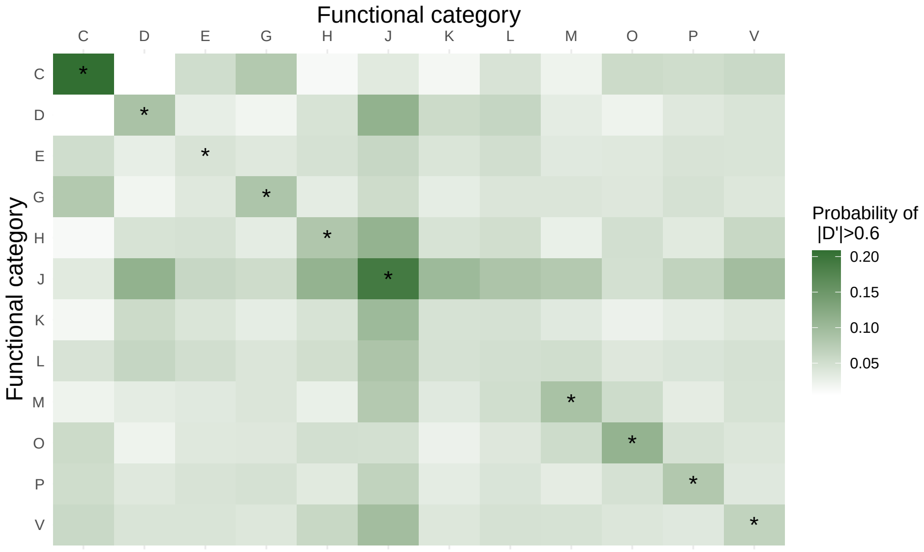

Fig R. LD patterns according to gene function for all pairs of genes. This figure reproduces the results of 4 in Main Text.a, without the 100kb distance threshold, therefore including pairs of genes very close on the chromosome.

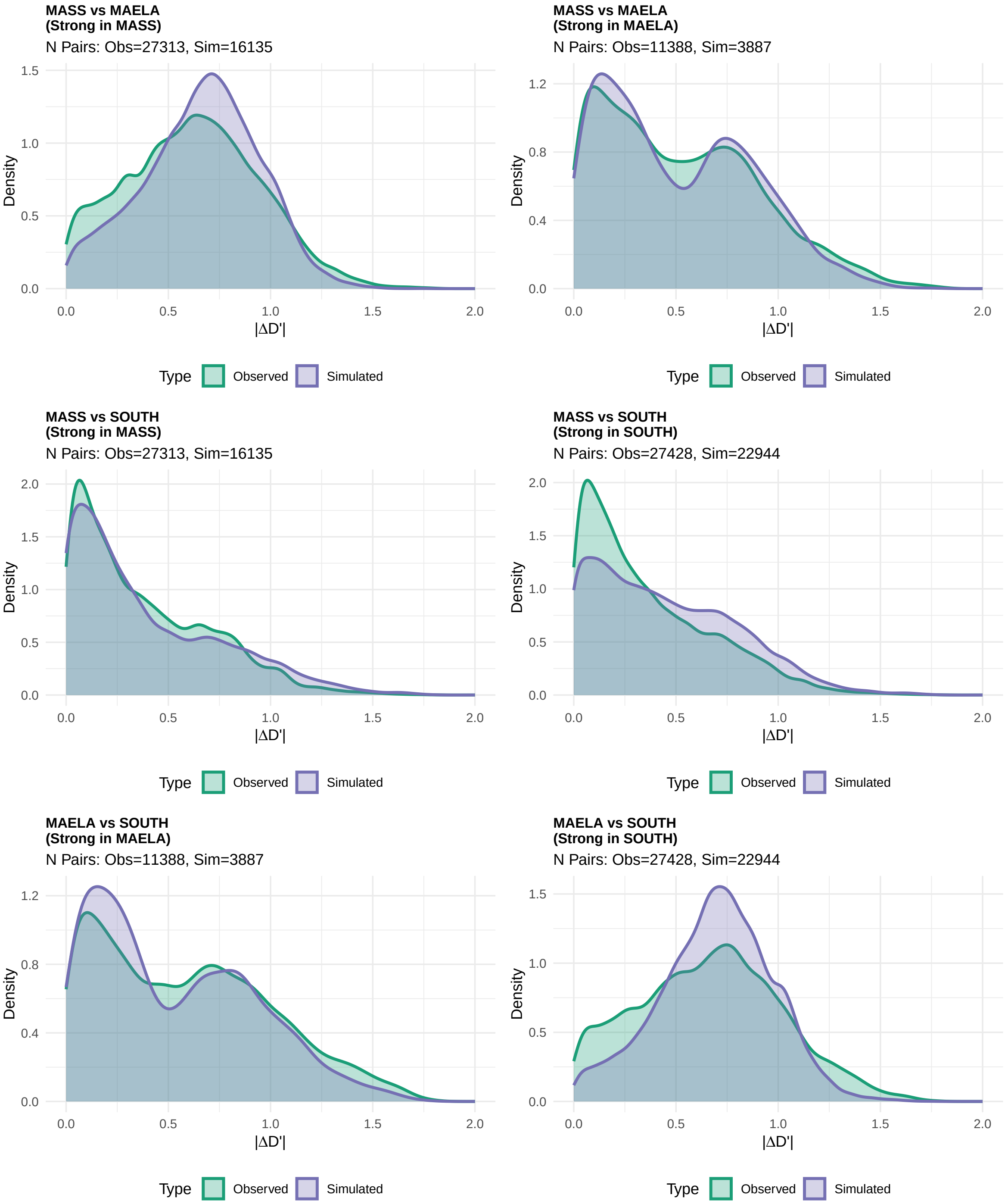

Fig S. Extension of main Fig 4 including all pairs of population, and all focal populations. For instance the first row compares the Massachusetts and MaeLa datasets. The left plot focuses on the fate of genes strongly linked in the Massachusetts dataset when observed in the MaeLa population. The plot on the right focuses on genes strongly linked in the MaeLa dataset and their fate in the Massachusetts population. The number of pairs making up each distribution are shown for both observed and simulated populations.

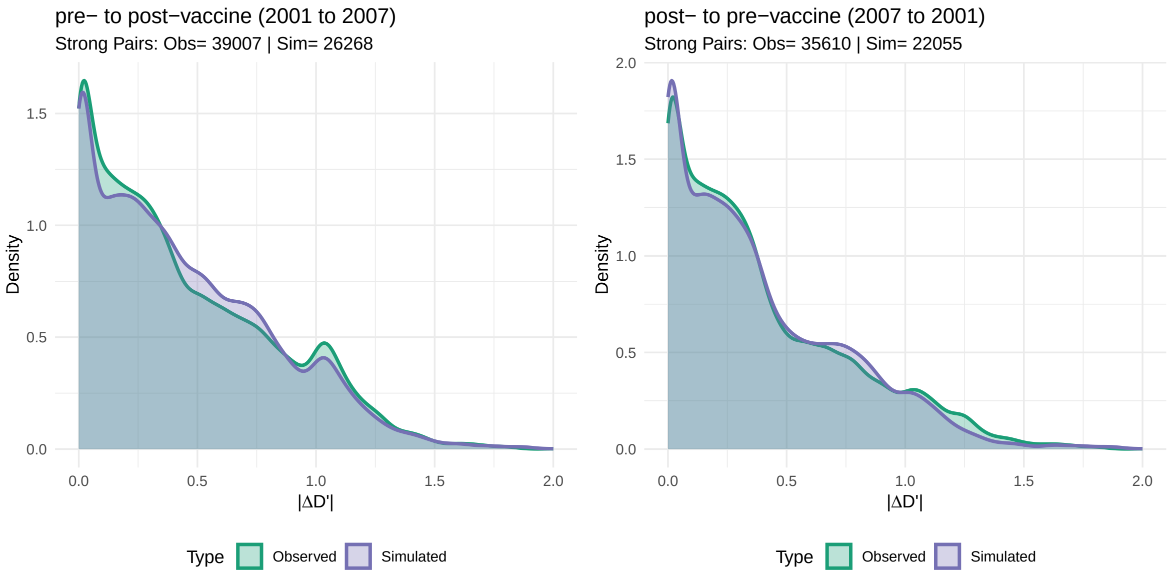

Fig T. Patterns of LD change across time in Massachusetts between 2001 (before vaccination) and 2007 (after vaccination) for each pair of gene with strong LD in the 2001 dataset. We study the distributions of absolute difference of LD across populations. We show for gene pairs highly linked in the 2001 dataset ($|D'| > 0.6$) the distribution of change in linkage compared to the 2007 dataset. The green line shows the distribution of observed changes, and the orange line shows the distribution of changes in the simulated dataset.

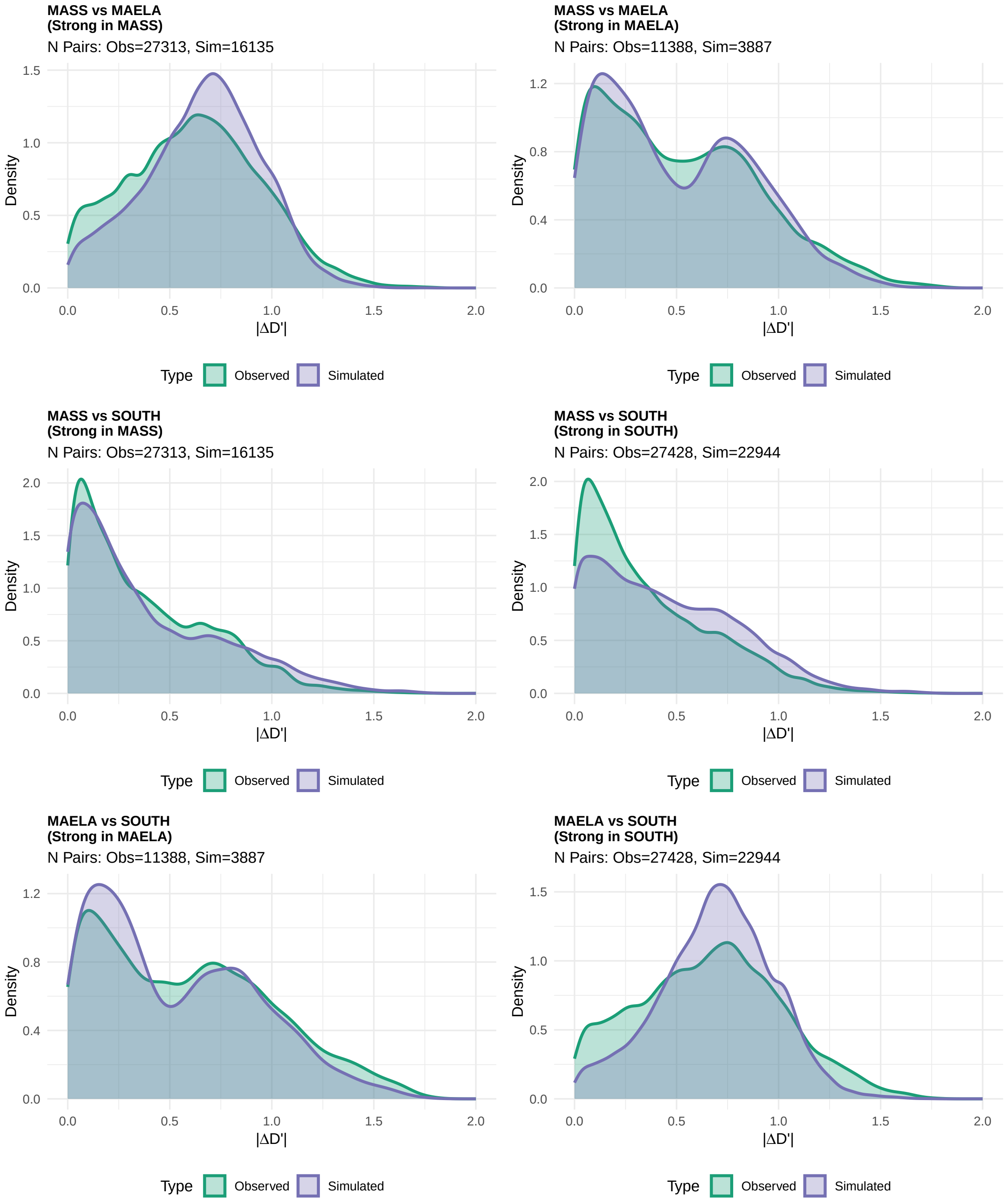

Fig U. Subsampling the populations to equalize number of isolates does not change LD change patterns across populations. This figure reproduces Fig S after subsampling the MaeLa and Massachusetts datasets to match the number of isolates in the smaller Southampton dataset

References

[1] Gabrielle L Harrow, John A Lees, William P Hanage, Marc Lipsitch, Jukka Corander, Caroline Colijn, and Nicholas J Croucher. Negative frequency-dependent selection and asymmetrical trans-

formation stabilise multi-strain bacterial population structures. *The ISME Journal*, 15(5):1523–1538, 2021.
