# Supplementary material for: Epistasis mediates the role of negative frequency-dependent selection in bacterial strain structure
Source: PLoS Comput Biol. 2026 Mar 19;22(3):e1014083. doi: 10.1371/journal.pcbi.1014083 (PMC13029793; doi:10.1371/journal.pcbi.1014083)
Supplement: Attachment — Submitted filename: PCompBiol Reviews.pdf [file pcbi.1014083.s002.pdf]

Reviewers' comments:

Reviewer's Responses to Questions
Reviewer #1: This manuscript by Guillemet & Lehtinen is an intriguing exploration of the interaction between epistasis and frequency-dependent selection on bacterial strain structure. This builds on previous work on McLeod et al (https://www.pnas.org/doi/10.1073/pnas.2401578121), which focused on immune selection. This manuscript extends the work to consider competition between commensals through two different mechanisms.

My major criticisms of the work are:

[1] The title and Discussion refer to "emergent epistasis". An emergent property is one that the system has, but the components do not. Yet in each case, the epistasis is assumed by the equations that define the model, rather than being inferred from observations of simulation results.

To clarify, the Discussion states, "there is no a priori reason to assume effects combine additively. Deviating from this assumption leads to emergent epistatic effects". Yet one of the original definitions of epistasis, by Fisher, is "any statistical deviation from the additive combination of two loci in their effects on a phenotype" (https://pmc.ncbi.nlm.nih.gov/articles/PMC2689140/#BX2). Therefore the epistasis is assumed, intrinsic to the equations, not emergent.

The results shown in Figure 3 are the closest to being emergent. However, in these simulations, the epistasis still requires that the effect of a two allele deviation is not equal to double the effect of a one allele deviation. This again is a violation of additivity, and therefore implicit epistasis within the equations that define the model.

The authors should therefore comment on how their work compares with previous analyses of the effect of epistasis on the emergence of strain structure in a recombining population (e.g. https://doi.org/10.1073/pnas.0812560106).

Thank you for this comment and the references. We agree that the use of "emergent" is not appropriate as epistasis is not an emergent property of our models but rather an explicit component. However if epistasis as a whole is not emergent, we wonder if you would agree that the *genotype frequency-dependent* aspect of the epistasis is an emerging property? We modified the text so that emergent/emerging is only used to describe this aspect of epistasis, notably we removed emergent from the title.

Thank you for the suggestion of Neher&Shraiman2009. We focus on epistasis between pairs of genes under NFDS, while Neher&Shraiman show how many pairs of genes with weak epistasis can lead to whole clusters of genes to be consistently under strong LD. This article is very relevant and informative in putting our findings in the context of whole genomes rather than simply pairwise interactions. We now discuss this in a paragraph (lines 410-416) at the end of the discussion.

[2] The validation of the model predictions using genomic data do not appear to consider physical linkage through spatial proximity of genes in a chromosome. The null distribution to which the biological data are compared was generated through shuffling the presence of genes across genomes, which is inconsistent with the known genome biology of these bacteria (e.g. the structuring of genes into operons), and their population structure (which is not panmictic). Therefore is it unsurprising that the observed distribution deviates from the null distribution. The authors should limit their comparisons to genes separated by a significant distance (e.g. 100 kb) to minimise the effects of

physical linkage.

The authors do sensibly limit their analysis to genes that are gained or lost at least 30 times on the tree. However, such rapidly moving genes are likely to be mobile genetic elements. These often represent blocks of many genes that, by definition, move as a single linked unit. Therefore it is to be expected that they will exhibit high LD. It is probable that these structures account for the lower-than-expected proportion of reversals observed following vaccine introduction. This explanation fits with the authors noting there are many integrases and transposes in the set of genes showing the highest linkage, “potentially reflecting the role of mobile genetic elements in LD”. This issue should also be fixed by using the distance threshold suggested above to only look at distantly-separated genes.

This is a great suggestion. It is however not trivial to apply a distance threshold because we are studying accessory genes which are by definition not always present, and when they are, the position on the genome is not necessarily the same in all isolates. The genomes we use are also not perfect assemblies but rather are divided into contigs (median=61 contigs per genome). We therefore approached this distance threshold, as follows:

-For each genome, we check for each pair of genes whether the following condition is true:
    (the two genes are on the same contig AND more than 100kb apart) OR (they are on different contigs)
-We only keep pairs of genes for which this condition is true in more than 99% of the genomes where they are present.

This results in keeping 76.07% of gene pairs. The new figures are now Figure S16 (conservation of linkage) and S17 (linkage according to functional annotation). This new filtering greatly limits the frequency of LD conservation in Figure S16, but has little impact on the absolute number of reversal events, therefore we find an even greater fraction of LD reversal.
Not surprisingly, the signal of observing more LD between genes with the same KEGG functional annotation is very diminished in Figure S17: likely, genes with similar functions have significant epistasis, therefore are clustered physically on the genome to prevent dissociation by recombination and are thus removed from the analysis. We discuss this in main text lines 344-352.
However, even with this threshold, the observed number of reversals between pre- and post-vaccine populations remain lower than expected by chance, see Table S3 and S4.

Additionally, if there are epistatic interactions between genes, why would selection not result in them being found in the same operon? At which point, would it not be expected that they would be co-inherited, and therefore cease to affect population structure?

We completely agree with the first sentence, at least when epistasis is rather strong. The goal of our work is to show how wide-spread such epistatic interactions could be in the accessory genome for genes under NFDS. If the epistasis is positive, this does make it more likely that the genes will be found together in a genetic cluster (cf. as in Neher&Shraiman 2009). However we do not quite understand why it would cease to affect population structure. Even with low recombination due to the physical proximity on the chromosome, these genes would still be maintained in high linkage by epistasis. Also if genes were locked together in this way, this would create a strong LD signal, and some lineages would have all of the involved genes, while the other would have none. This leads to non-random associations between alleles, i.e. strain structure.

[3] Equation 1 features “the allele frequency favored by selection” for each allele, which features in the

fitness calculations. However, these do not appear to be mentioned again in the paper. Figure 2b suggests that all four favored frequencies are 0.5. Is this the case? Do all the equations generalize to any favored frequency? What effect do unequal frequencies have?

It is indeed the case that in the base metabolic competition model, NFDS drives all allele frequencies to the same frequencies, ie. 0.5 at equilibrium. We added a sentence in line 166-168 to better convey this.

To explore the effect of deviating from this equilibrium frequency of 0.5 in the metabolic competition model, we added a new Supplementary Note 2: Asymmetric metabolic loci. To change equilibrium frequencies, we use an additive transmission benefit to one allele per locus. The impact on equilibrium frequencies and LD is explored in FigureS8 and we find that our previous conclusions are robust to this change. The only difference we observe is when allele level selection is strong enough to cause the least fit genotype to be completely absent at equilibrium, resulting in negative equilibrium LD even when PFDS would push for a positive equilibrium LD. These additional results are now referenced in lines 207-210.

In contrast, in the competition-colonisation model, the equilibrium frequencies are not necessarily 0.5, as this depends on the interaction between the relative efficiency of co-colonisation q, the colonisation benefit m and the competition parameters kΔ. The variation in equilibrium frequency is explored in Figure S10. We added a sentence in main line 248-250 to emphasize this emerging equilibrium frequency.

Additionally, it would be reassuring to see the framework used to undertake neutral simulations, with the results included in the SOM for comparison with the NFDS simulations.

This is indeed important to check. We now implement a neutral version of the metabolic model (no NFDS) in Figure S2, using k_0=k_1=k_2=1, and three different starting conditions, showing that LD and allele frequencies remain constant in that case. This is referenced in the main text lines 169-172.

My minor comments are:

[a] Equation (3) – the top equation only applies when fitneses are additive, according to McLeod et al (page 6, SOM) – this should be clarified
We added a precision: “**additive** selection coefficient”. However it is not exactly that fitness values are additive which leads to this: it is rather that selection coefficients are usually modeled as additive when in continuous time (as opposed to multiplicative in discrete time) to prevent built-in epistasis. Still, it is a source of confusion in the field (see Mcleod&Gandon 2021, Box 1 https://elifesciences.org/articles/65645) and we thank you for noting this, hopefully, adding the word “additive” and adding this reference should reduce confusion.

[b] Equation (4) – does not match equation (16), or McLeod et al SOM equation 22c – can the authors explain this discrepancy?

We tried to simplify some of the results for the main text to highlight the role of epistasis: in this case the missing term in our Equation (4) compared to McLeod et al. vanishes when the individual selection coefficients of each allele are zero. In an effort to make the manuscript more accessible, we used this assumption to simplify this equation, highlighting the main effect that epistasis drives LD

dynamics. We reconsidered this choice and now provide the same equation as in McLeod2024. We added a sentence below (lines 129-131) to note how the first term (which we initially did not show) vanishes when individual selection coefficients go to zero, ie. when their respective equilibrium frequencies are reached.

[c] L129 – sigma not defined here, only in Methods
We now define sigma (line 136) as the recombination rate and better point to the corresponding method section.

[d] Figure 2 – why is there an infinity sign next to D' in the scale? (see also Fig. S6) Also, the zero of beta_0 is not subscript in many figure legends.
The notation was replaced with D_eq in all cases.

[e] L256 – "D'egative"
Fixed, was meant to be "equilibrium D' **for the n**egative frequency-dependent epistasis case"

[f] L312 – there is no Figure 4d.
Now correctly points to Figure S12.

[g] Equation 9 – "B" should be subscript
Fixed.

[h] L611-613: "bacteria" is plural
Plural is now consistent in the sentence

[i] Figure S3 – should be "null or negative"
Fixed

[j] The graphs in panel a of Figure S7 and S8 appear to be misformatted.
We have remade panel a, attempting to make it clearer.

[j] "Streptococcuc" in the keywords
We will fix this at resubmission through the manuscript portal.

Thank you for catching all of these.

Reviewer #2: Summary:
This manuscript explores how multi-locus negative frequency-dependent selection (NFDS) generates emergent epistasis, thereby shaping bacterial strain structure. Using two mechanistic epidemiological models (metabolic niche differentiation and competition–colonisation trade-offs), coupled with genomic analysis of >3000 Streptococcus pneumoniae genomes, the authors argue that frequency-dependent epistasis explains patterns of linkage disequilibrium (LD). The study aims to generalise beyond antigen-driven NFDS to broader mechanisms.
The study is conceptually rich, methodologically sophisticated, and relevant to evolutionary epidemiology. However, critical issues limit its clarity and robustness:
1. Insufficient justification of model assumptions (e.g., additivity vs. non-additivity of NFDS).
2. Ambiguity in parameter choices and simulation setups (e.g., competition parameters k values, recombination rates, initial genotype frequencies).
3. Limited exploration of alternative explanations for LD patterns (e.g., demographic effects, hitchhiking, clonality).

4. Data analysis lacks transparency in statistical testing, null model assumptions, and robustness across populations.
5. Overinterpretation: the empirical results are suggestive but not conclusive about emergent epistasis.

Major Critiques:
1. Abstract: Claims strong support for emergent epistasis from genomic data, yet the empirical evidence is largely correlative. This overstates certainty.
We agree with the comments and tempered the conclusions, eg. “multi-locus NFDS shapes bacterial strain structure” was changed to “**can** shape”. We also added the precision that we generalise and extend previous **theoretical** work on antigen-specific immunity.

2. Introduction: While antigen-specific immunity is mentioned, the transition to metabolic and competition–colonisation models feels abrupt. The rationale for focusing on these two mechanisms should be justified more strongly with literature. Moreover, the introduction does not sufficiently discuss alternative balancing mechanisms (e.g., fluctuating selection, spatial heterogeneity, migration).

We agree with this was not sufficiently explained, thank you for pointing this out. We focus on the within-host niche and competition-colonisation models as these are the mechanisms, along with strain-specific immunity, through which NFDS has been reported to arise in epidemiological models. This of course does not mean other mechanisms are not possible. We now discuss this in more detail in the introduction and also expand on non-NFDS balancing mechanisms (lines 70-77).

3. Methods, Epidemiological Models: Model assumptions are critical but under-justified. Why assume symmetric bi-allelic loci? Why ignore asymmetry in gene gain vs. loss (noted later in Supplementary but not addressed in main text)? Equations (5) and (8): Terms governing epistasis depend sensitively on parameter geometry (k0, k1, k2, kΔ). How were these parameter ranges chosen? Are they biologically realistic? Moreover, recombination treatment (σ terms) is simplistic. Empirical pneumococcal recombination is asymmetric, biased toward deletions. The model omits this key factor despite its known importance.

First and related to the reviewer’s next comment as well, we added Table 1 to list parameters with their dimensions and value ranges used throughout the manuscript. The epidemiological parameters correspond to a basic reproduction number of 3 (in the plausible range for *S. pneumoniae*). However, the specific values are not important: the key results relating the k parameters to the effect on strain structure (Equations 5 and 8) do not depend on the epidemiological parameters. The bounds on the k parameters (0,1) arise from the assumption that co-colonisation is not as efficient as primary colonisation, in line with empirical findings (e.g. Krishna et al. Plos Biology, 2025).

We now clarify this in the legend of the new parameter table.

Regarding the relationship between the k-terms in the two mechanistic models, these arise from the modelled processes: the efficiency of co-colonisation decreases with the number of shared alleles (metabolic model) or increases with difference in number of competitive alleles between the resident and incoming strain (competition-colonisation model).
Regarding recombination asymmetry, we do not include this in the main model because we are interested in exploring the effect NFDS has, by itself, on strain structure. Including asymmetric recombination would make the results more difficult to interpret, as it would then be unclear whether

observed effects were due to the mechanism we describe or the mechanism described in Harrow et al.

We now explore the combined effect of the mechanism we describe and asymmetric recombination in a new figure S9 and comment on it in the main text(lines 210-213).

Phenomenological baseline (Eq. 1): assumptions, units, regime of validity: Eq. (1) adds one-locus NFDS terms to logistic growth to show that with recombination there is no sustained LD because $w_{AB} = 0$ at the allele optimum. However, the conditions under which Eq. (1) approximates the mechanistic models (weak selection, quasi-linkage equilibrium, additive fitness, timescale separation) are not stated. Please add an explicit 'Assumptions & Units' paragraph and a parameter table (confirm r, ρ have units 1/time; p is unitless).

We are not sure whether we fully understand the reviewer's comment here. The purpose of this section (apart from building intuition and introducing terminology) is to demonstrate that when NFDS combines additively, there is no LD. The introduction of the explicit mechanistic models is motivated by the fact that it is unclear what the assumption of additive NFDS means biologically (lines 141).

We think the reviewer is saying that the result that additive NFDS leads to LD = 0 from the phenomenological model only applies to the mechanistic models under certain assumptions (e.g. weak selection). However, this is not the case, as these assumptions are not necessary for deriving the model.

We added Table 1:List of parameters and their dimensions.

4. Results, Simulation Evidence: Figure 2b/2c & Figure 3b/3c: The authors claim PFDS vs. NFDS regimes emerge depending on parameter geometry, but it is unclear how initial genotype frequencies ($D' = 0.4$) were selected. Different starting conditions may bias toward observing bistability. In addition, figure S7/S8: Exploration of parameter space is extensive, but the biological interpretation of "concavity" and "step" parameters is not intuitive. The authors should connect these meta-parameters explicitly to measurable biological quantities and this earlier on in the texts for benefit of readership…

Regarding the first point, we detail LD trajectories in Figure S6 and we added more intermediate initial values of LD. We also added FigureS7 reproducing Figure S6 but with different starting allele frequencies, $f_A=f_B=0.1$, to show the robustness of the trajectories. There are exceptions for some specific cases (additive case without recombination, and reinforcing PFDS with initial LD close to zero) which we describe in lines 220-224..

We have rephrased the results to make the effect of convexity or concavity more intuitive, by noting that it results respectively in a synergistic interaction, or an antagonistic/saturating interaction between competitive alleles. (lines261-264). The geometries leading to frequency-dependent effects are harder to interpret biologically as they refer to everything that is not strictly the two previous cases (ie. concave=always saturating or convex=always synergistic). We try to make this clearer in the next paragraph (lines 272-276).

We have also rephrased the definitions of the concavity and step parameter in the Supplementary (lines 744-751). It is worth noting that we introduce these meta-parameters in order to explore and visualise the model's behaviour in parameter space. While the meta-parameters are tricky to interpret and measure, the k parameters themselves have clear interpretations and are measurable.

5. Results, Genomic Data Analysis: LD analysis is interesting but the statistical testing is insufficiently detailed. The permutation null assumes average LD = 0, which is unrealistic under recombination–selection balance. More sophisticated nulls (e.g., coalescent simulations incorporating recombination rates, clonality, demography) should be considered. Moreover, the evidence for LD sign reversals (PFDS) is rare (2.7~4.5% of pairs?). Yet this is central to the argument. The rarity of reversals suggests that constant epistasis and demographic structure may still explain most observations.

We agree that the model we use is not nearly realistic enough to model the evolution of a species such as *S. pneumoniae*, and it is not our goal here to do so. Building a “realistic” null model is extremely challenging and even just its parametrisation would probably be impossible with our current understanding of the population dynamics of *S. pneumoniae.* We now discuss this in lines 417-422. What we aim to do is, following the recent observations showing that many genes are maintained by NFDS, show that this could lead to wide-spread epistasis, that can potentially be positive or negative frequency-dependent.

We then want to illustrate this question by exploring a dataset of *S. pneumoniae* genomes. We do find some results that are consistent with the mechanism we propose (and notably some, reversal events, that are not consistent with other mechanisms). We do not propose that they are the only drivers of observed patterns. We do try to limit as much as possible the influence of other effects:

-clonality: we used ancestral reconstruction to infer the gene content of internal nodes of the *S. pneumoniae* tree, and we removed from the analysis genes that have been gained and lost less than 30 times. This ensures that observed LD is not due to genes simply being borne by one or a few very successful clones which are now the ancestors of whole lineages. We added emphasis on this point in the results and discussion sections. (lines 309-316)

-mobile genetic elements: we now restrict our analysis to genes distant of at least 100kb on the chromosome in Figure S16 and S17, see comments from and response to reviewer#1).

Regarding the number of reversal events, we agree that this is not a very large number of pairs exhibiting reversals but first, even our modeling results suggest that these reversal events should be rare. They should only happen when two conditions are met: the selection parameters lead to epistasis being positive-frequency-dependent (reinforcing current LD), and a strong enough perturbation which is able to disrupt the LD to make it jump to the other equilibrium (eg. from strong negative to positive).
Second, the populations seem to be quite mixed as we see in Figure S13.a that the different locations are dispersed on the phylogenetic tree. The proximity of these populations make it so that comparing them is maybe not the “strong” perturbation which we mention in the first point.

6. Discussion: The discussion overstates the empirical support for frequency-dependent epistasis. At minimum, claims should be tempered: the data are "consistent with" rather than "demonstrating" the mechanism. Also, while limitations are acknowledged, they are not sufficiently addressed. For example:

We agree with the point about tempering language and have adapted the wording throughout the discussion, eg.:

-suggesting these associations ~~are~~ **could be** driven by epistatic rather

~~Here, we show that NFDS, which gives rise to the former, also impacts the latter through emergent epistasis.~~
Here, we show that NFDS, which gives rise to the former, **can** also impact the latter through epistasis.

a. No discussion of recombination rate heterogeneity across populations.
We now mention this in the discussion (line 420), although we are not certain what the expected effect or its size would be.

b. Lack of validation against longitudinal data beyond the Massachusetts vaccine rollout.
We agree that this is only a single example of longitudinal data, making it difficult to draw general conclusions. We added a sentence to the discussion to address this. (lines 428-430)

c. No comparison to simpler explanations such as persistent clonal structure.
We tried as much as possible to limit the impact of clonal structure by removing genes that have rarely been gained and lost in the evolutionary history of the species. Yet we agree that this is still a major contributor to strain structure. We now emphasise these other explaining factors in the discussion (lines 417-422).

7. Overall Replicability: The ODE systems are detailed, but the parameterization is not fully transparent. Supplementary material provides equations but not always parameter ranges or biological justification. Furthermore, the Zenodo link to the cited code appears broken, making it impossible to verify or reproduce the simulation workflow directly. As a result, the manuscript text alone does not provide sufficient information to ensure full reproducibility without access to external files.

We are sorry for the accessibility to the zenodo repository, indeed simply looking for the DOI (although the correct one) did not appear to lead to the repository. We have modified the manuscript to include the direct and clickable link instead of the DOI.

We have also provided a table summarising parameter dimension, ranges and biological justifications.

Minor Critiques:
1. Abstract, "emerging epistasis … unlike classical epistasis" Needs clearer definition; the distinction between frequency-dependent and classical epistasis is not well explained.
Thank you for the suggestion, we have added the precision “constant sign” when referring to “classical epistasis”.

2. Results, Figure 2 captions: Ambiguity in describing "competition efficiencies"—should be defined consistently.
To make this more consistent, we have added “competition” before “efficiency” every time we refer to the k0 k1 or k2 parameters (eg. when we first define them line 159).

3. Methods: Clarify whether allele frequencies were initialized deterministically or stochastically in simulations.
We apologise for the oversight, we indeed used deterministic initial allele frequencies of $p_A=p_B=0.5$ throughout the manuscript. This is now stated in the legend of Figure 2. Note that we did not use 0.5 because this can lead to numerical inconsistencies, for instance if frequencies are pushed by selection to 0 if <0.5 and 1 if >0.5. However, initial frequencies do not change the equilibria in our work as long as all alleles are present initially. We now show this in Figure S7 with starting allele frequencies of $f_A=f_B=0.1$, except for some specific cases described in a response to comment 4.

4. The main text should summarize key points instead of relegating critical assumptions (like recombination asymmetry) to Supplementary.
We now address recombination asymmetry and allele fitness asymmetry in the main text (lines 207-213).

Issue of Grammar, Spelling, Proofreading, etc.
1. Generally well written, but several sentences are long and convoluted (e.g., Discussion pp. 13–14). Shorter, more direct sentences would aid readability.
We have divided six (very) long sentences into shorter sentences throughout the discussion.

2. Inconsistent hyphenation: “co-colonisation” vs. “co colonisation”.
We are now consistent with the hyphenation and all instances have been changed to ‘co-colonisation’.

3. Abbreviation NFDS/PFDS should be redefined in the Discussion for clarity to non-specialist readers.
We redefined NFDS in the discussion (we don’t refer to PFDS in the revised discussion).

4. Typo in Eq. 9.
Thanks, fixed.

Reviewer #3: This manuscript addresses how multilocus negative frequency-dependent selection (NFDS) can generate emergent epistasis that, in turn, structures bacterial populations into strains. By analyzing two mechanistic models (metabolic niches and competition–colonization trade-offs), the authors show that both frequency-independent and frequency-dependent forms of epistasis can arise, including positive frequency-dependent selection (PFDS). They then test these predictions with large-scale genomic data using linkage disequilibrium (LD) analyses of accessory genes. The study is ambitious, broad in scope, and effectively bridges theoretical and empirical approaches, making it well suited for PLOS Computational Biology.

Major Comments
In the analysis of large-scale genomic data of S. pneumoniae, the identification of PFDS relies on detecting LD sign reversals across populations, under the assumption that stationary epistasis would not lead to such reversals. However, other processes (e.g., lineage-specific recombination rates, mobile genetic elements, historical bottlenecks/founder events) could also generate sign reversals. The authors should explain in greater detail how these alternative mechanisms can be excluded.
The permutation scheme shuffles gene presence/absence across strains while preserving frequencies, but this procedure destroys phylogenetic structure and signals of clonal expansion. Alternative null models—such as block permutations within lineages or phylogeny-based simulations (e.g., PAST-ML–consistent evolutionary histories)—should be considered.
While the manuscript addresses an interesting and timely question, I find the current evidence insufficient to support the central claim that PFDS and NFDS emergently structure bacterial strain diversity. The modeling framework is overly simplified to capture the complexity of pneumococcal accessory genome dynamics, and the empirical LD analyses are heavily confounded by MGEs and population structure. The inference of PFDS from rare LD reversals is particularly weak and may reflect artifacts of the null model rather than true biological signals. A more refined discussion of the gap between theoretical models and empirical data is needed.

We thank the reviewer for these comments. We agree that the mathematical model we use is not nearly realistic enough to fully model capture pneumococcal accessory genome dynamics. Our aim is to generate qualitative insights about a potential mechanism shaping these dynamics, rather than fully

capturing their complexity. We have reworded the manuscript to make this clearer, particularly in the discussion (eg. lines 363, 417-422).

We also agree that the null model is very simple and does not account for multiple alternative mechanisms. However, parametrising a more realistic null model is not possible, because we cannot quantify how much alternative mechanisms contribute to observed patterns of LD. However, we seek to control for alternative mechanisms in the following ways:

-clonality: we used ancestral reconstruction to infer the gene content of internal nodes of the *S. pneumoniae* tree, and we removed from the analysis genes that have been gained and lost less than 30 times. This ensures that observed LD is not due to genes simply being borne by one or a few very successful clones which are now the ancestors of whole lineages. We added emphasis on this point in the results. (lines 309-316).

-mobile genetic elements: we added an sensitivity check in which we restrict the analysis to genes distant of at least 100kb on the chromosome in Figure S16 and S17, see comments from and response to reviewer#1).

We now discuss these points, as well as the limitations to the interpretation of the empirical analysis, in more depth in the discussion (lines 417-422).

Thank you for the suggestion of within-lineage permutations. After reflection, we do not think this is an appropriate null model in our case. To illustrate this, consider a species with ideal strain structure: in this case, all gene pairs would be either at maximum LD or minimum LD, and therefore all the isolates within a given sequence cluster would share an identical accessory gene content. In that case, because of the non-existent within sequence cluster diversity, the reshuffling strategy would have zero impact, leading to observed and expected LD change distributions being identical. Therefore, the reshuffling strategy would erase the very signal that we are interested in.

We also now discuss our findings in relation to the work of Shraiman&Neher 2009 (lines 410-416 and see response to reviewer#1), where the authors showed how many genes with even weak epistasis could lead to clustering on the chromosome and conserved linkage. We believe our findings propose a plausible mechanism for the presence of such wide-spread epistasis in the accessory genome.

Finally we added a paragraph to stress the fact that we do not propose that the mechanism we explore in this work is not the only mechanism shaping strain structure, and we acknowledge the contribution of other mechanisms such as clonality, demographic events, recombination heterogeneity, and spatial structure - and the role of more complex models in quantifying the relative importance of these different processes.

Minor Comments
i) There appear to be discrepancies between figure references and the text. For example, in line 175, the reference to “(Figure 2a, dashed line)” seems to correspond to the red line rather than a dashed one.
This is now fixed, thank you for catching that.

ii) In line 312, the reference to “(Figure 4d)” does not correspond to an existing figure.
This now correctly points to Figure S12.
