# Supplementary material for: Epistasis mediates the role of negative frequency-dependent selection in bacterial strain structure
Source: PLoS Comput Biol. 2026 Mar 19;22(3):e1014083. doi: 10.1371/journal.pcbi.1014083 (PMC13029793; doi:10.1371/journal.pcbi.1014083)
Supplement: Attachment — Submitted filename: Revisions_2.pdf [file pcbi.1014083.s003.pdf]

We thank the reviewer for their thoughtful comments, and in particular, for picking up on the issues with the null model we were using. We very much appreciate the time the reviewer took both to think about the issue and to explain their reasoning. We think the manuscript is much improved by the suggestions.

We agree with the reviewer's concern about the original null model and most of the reviewer's comments more generally. Our full response is below, outlining changes to the manuscript and our points of disagreement with the reviewer (which are minor). In brief:

- We generally agree with the reviewer's reasoning, with the nuance that we did not intend to make strong claims about LD conservation, or effects being widespread. LD conservation is consistent with both constant-sign and frequency-dependent epistatic effects. We are more interested in LD reversal, because this is only consistent with the frequency-dependent effects.
- We implemented the null model suggested by the reviewer and revised the results and discussion accordingly. The evidence for LD conservation using this null model is mixed (see comments below), but we see clear evidence for LD reversal.

Reviewers' comments:

Reviewer's Responses to Questions

Comments to the Authors:

Reviewer #1: [1] The authors ask, "we wonder if you would agree that the genotype frequency-dependent aspect of the epistasis is an emerging property?" I think this is reasonable, as it emerges from an interaction in the simulations, not an explicit definition within the model.

Thank you for this comment

[2] I thank the authors for introducing the correction for physical linkage shown in Figures S16 and S17. These figures should replace Fig. 4a and 4b, because the comparison demonstrates that the vast majority of gene pairs showing consistent linkage between populations are close together, which is not relevant to the effect of epistasis on population structure. Similarly, S15 should be dropped, and S18 recalculated using the distance thresholds. Only these figures that incorporate the physical distance thresholds enable the reasoning below, which is detailed such that the authors can correct me if I have misunderstood:

We have now removed all the figures that did not incorporate 100kb filtering. The only exception is the previous Figure 4a which we have now moved to the Supplementary (S18). We think it is reassuring to see the LD patterns according to functional annotation, as it does show a strong D' for genes of shared functions which also tend to be close on the genomes (eg. operons).

(a) The authors state in the manuscript "the strong linkage is conserved across populations (defined as |D'| > 0.6 and same sign LD) for 30.6% of gene pairs" (this should be updated in a revision). In Fig. 4b, the shuffled population has a mode around 0.6, which is consistent with there being zero LD, as expected in these permuted data (the comparison is between D'> 0.6 in one population, and D ~ 0 in the shuffled data). The mass of smaller delta D' values in the observed data was used as evidence of widespread epistasis in the original submission.

Yes, although we would like to nuance this a little.

- We did not intend to make strong claims about LD conservation in the manuscript: LD conservation is consistent with both constant-sign and frequency-dependent epistatic effects. We are more interested in LD reversal, because this is only consistent with the frequency-dependent effects. We have further clarified this in the revised manuscript.
- We did not intend to make claims about epistasis being widespread. We explicitly point out that estimating the role of these effects in practice is not feasible. We have further clarified this in the revised manuscript.

(b) However, 23.9% of gene pairs have to be removed through close physical linkage – similar to the proportion that exhibit strong linkage conservation across populations. Accordingly, Fig. S16 shows that the mode for the observed data delta D' is also now 0.6 – that is, strong linkage pairs in the focal population are generally not linked in the comparator population. Therefore linkage that is observed in one population is not apparent in another. This is not consistent with widespread stable epistasis across populations. This should be made explicit.
(c) Nevertheless, the authors claim that Table S3 (note this table has this symbol in the legend: "¿100 kb") shows that there are significantly more LD conservation events than expected between populations. This is based on the tail of small delta D values in Fig. S16, that lie outside of the narrower shuffled population distribution used as the null. However, the reason for this difference is not a difference in the position of the mode, it is a difference in the variance of the null and observed distributions. The larger variance of the observed data is due to the effects of population structure that are not accounted for in the permutations: permutation treats each isolate as an individual sample, rather than one that shares gene content through ancestry with other isolates. Hence the effective sample size of the observed population is smaller than the sample size of the shuffled population (i.e. why effective population sizes are smaller than census population sizes). Therefore the variance of the null distribution shrinks relative to the observed population.

Yes, we agree. Thank you very much for noticing this.
(d) The problem this creates is best illustrated with the Maela-Southampton comparison (Table S3):
(i) There are three time more strong LD pairs in the Maela population than the Southampton one
(ii) There are the same number of observed conservation events in the comparison between the two (which is necessarily the case)
(iii) The number of expected conservation events is 2,500-fold higher for the Southampton-Maela comparison than the Maela-Southampton comparison, despite there being more strong LD pairs in Maela.
(iv) A comparison with Table S1 shows that these differences are not down to chance fluctuations in the permutation distribution – there is a 1,600-fold difference in the expected numbers for the Southampton-Maela comparison compared to the Maela-Southampton in that analysis as well
(v) After trying to work this out for some time, I found the size of the sample from Maela is much larger than the size of the sample from Southampton (https://microreact.org/project/multilocusNFDS) – this information should be reported in the manuscript – narrowing the variance of the null distribution, resulting in a reduced number of expected conservation events.
(vi) Yet Fig. S16 shows the observed delta D' distribution is approximately consistent across all population comparisons, regardless of sample size – indicating that the difference between the observed and expected values should not be strongly affected by sample size, if the comparison were appropriately calibrated
(e) This also affects the number of reversals (Table S4), which corresponds to the tail of delta D' values >1.2. The expected number is again lowest in the comparisons using the Maela population as the focus, suggesting the effects are driven by sample size, rather than epistasis.

To answer these comments, we now redid the analysis with the new null model as suggested below. Regarding the differences in number of isolates between populations, we added Figure S21 which shows the D' changes between populations after subsampling the two biggest datasets (MaeLa and

Massachusetts) down to the isolate number of the Southampton dataset. We also now indicate the number of isolates for each population in the methods (lines 552)

The authors claim that their use of genes that have been gained or lost 30 times or more will eliminate the effects of clonality. However, https://microreact.org/project/multilocusNFDS shows there are >70 Sequence Clusters, so a gene could easily be conserved within half of these (and absent from the other half), while also being gained/lost more than 30 times. For a more convincing statistical analysis:
(i) Sequence clusters observed in both compared populations (e.g. Sequence Cluster 18, found in all three) should be dropped from the comparisons, as their presence makes populations similar through common ancestry
(ii) The observed distribution of gain/loss numbers should be shown to justify the selected threshold (or it should be adjusted, as appropriate)
(iii) Null simulations should be used that replicate this gain/loss distribution – this can be done using the tree and population genetics simulation software (e.g. https://academic.oup.com/sysbio/article/65/2/334/2427219)
(iv) D' values from these null simulations (with genes having similar gain/loss distributions in the observed and simulated populations) can then be compared with the observed ones as in the current manuscript

All these problems also affect the pre/post vaccine analysis of the Massachusetts population. The current version is making claims that simply cannot be robustly justified (e.g. much of the Discussion). While I appreciate the authors have added some caveats, they are not sufficient to highlight the problems to many readers with a biological background.

We agree with these points.

We now use a null model based on the suggested approach. The proposed software did not work for our dataset (our guess is that there are too many isolates on the tree). Instead we used the "castor" R package[1]. We calibrate the model to reproduce the observed gene frequencies (Figure S14) and the observed median parsimony scores (number of changes, Figure S15). The resulting simulated D' distributions are shown in Figure S16, and we show as an example the comparison between gene distributions in the observed and simulated Southampton population in Figure S17. The new figures comparing D' across populations in the observed data and the null model are in 4b.c, S18 and S19, and the updated statistical testing for conservation and reversal events are now in Table 1 and 2.

The two points where we did not follow the reviewer's recommendation:

-(i) Now that we have a more robust null model that incorporates phylogeny, we do not believe it is necessary to remove shared sequence clusters. There are more sequence clusters that are shared across populations and we would need an arbitrary threshold of what degree of sharing we consider as too much. Furthermore, we think removing shared sequence clusters would bias the results towards observing LD reversal. As we are most interested in the presence of LD reversal, we prefer to be conservative on this point.

-(ii) For the same reason, because we have a phylogenetically aware null model, we have now removed the filtering based on the number of gains and losses, as genes with few changes on the tree will also have few changes in the simulated dataset. This also removes an arbitrary threshold that we believe is now unnecessary.

We have updated the results and discussion sections to be in line with the new null model and results.

1. Stilianos Louca and Michael Doebeli. ‘Efficient comparative phylogenetics on large trees’. In: 710 Bioinformatics 34.6 (2018), pp. 1053–1055

Additionally, what is the explanation for the atypically strong associations seen with Translation genes in Fig 4a/S17? Is this down to a small number of genes, as translation machinery should rarely be in the accessory genome?

We mention in line 329-330 that genes from the functional category “J: Translation” include integrases and transposases and therefore likely point to mobile genetic elements. These still showing after the 100kb filtering could be due to mobile genetic elements clustering in hotspots where LD would be strong?

[3] Thank you for these additions – Fig. S2 needs a legend.

Thank you for pointing this out. We have added a legend for the different genotypes.

Reviewer #2: The comments and concerns raised have been addressed.

Reviewer #3: The authors have satisfactorily addressed all previous comments and concerns. The revisions have significantly improved the clarity and quality of the manuscript. I am satisfied with the current version and recommend acceptance in its present form.
