# Supplementary material for: Epistasis mediates the role of negative frequency-dependent selection in bacterial strain structure
Source: PLoS Comput Biol. 2026 Mar 19;22(3):e1014083. doi: 10.1371/journal.pcbi.1014083 (PMC13029793; doi:10.1371/journal.pcbi.1014083)
Supplement: Attachment — Submitted filename: response_reviewers_3.pdf [file pcbi.1014083.s004.pdf]

Reviewer #1: I thank the authors for their patience, and the changes they have made to the analysis, which address my concerns.

My only remaining comments regard the presentation of the Results. The authors state that “The evidence for LD conservation is less clear (Table 2): we find patterns of LD being both more and less conserved than expected by chance.” As it is unlikely that simulations can exactly replicate the observed level of LD, this result essentially implies that the observed level is indistinguishable from that expected by chance, and therefore there is no significant evidence of LD.

The authors state in the Results, “This filtering is likely to remove some pairs of genes in strong positive epistasis (e.g. genes within the same operon),” then in the Discussion they state, “the removal of physically close gene pairs underestimates the extent of positive epistasis.” Similarly, elsewhere in the revised Results it is stated that “the distance-based filtering we use to limit the impact of physical linkage is very likely to filter out gene pairs in strong positive LD, which would contribute to the lack of conservation we observe.”

However, such short-range interactions do not affect the structure of the population, because each of the genes will be co-transferred by individual horizontal gene transfer events. The authors themselves acknowledge this in the Introduction, which states, “Epistasis refers to the interaction between genes at different loci.”

The authors should change the Results and Discussion to reflect the lack of evidence for classical epistasis shaping the population structure, as there is no evidence for long-range epistasis being conserved across populations.

We agree that we have not been clear enough in expressing the evidence for a lack of constant sign epistasis for long ranges pairs. We have rephrased the results paragraph l352-358, and discussion paragraph l400-405 accordingly. We now highlight that we do not find evidence for constant sign epistasis being able to maintain long range allelic associations.

Additionally, in the Abstract, it is stated, “make observations consistent with epistatic effects on gene associations”. This would be interpreted by a casual reader as evidence of classical epistasis. It would be helpful to clarify the findings by editing the statement to “make observations consistent with frequency-dependent epistatic effects on gene associations.”

We edited the abstract as proposed, as it makes the abstract consistent with the previously discussed changes.

One additional comment: the text refers to “clonal genealogies.” However, it is not clear that recombination could have been removed when constructing such a

phylogeny, so I suspect it is not “clonal,” but an approximation from an alignment of recombining genotypes.

Thank you for this precision, we edited the text to replace ‘clonal genealogies’ with ‘inferred core genomes phylogenies’.
